# Sparse Density Estimation with Measurement Errors

**DOI:** 10.3390/e24010030

**Published:** 2021-12-24

**Authors:** Xiaowei Yang, Huiming Zhang, Haoyu Wei, Shouzheng Zhang

**Affiliations:** 1 School of Mathematics and Statistics, Chaohu University, Hefei 238000, China; yxw8290@163.com; 2 Department of Mathematics, Faculty of Science and Technology, University of Macau, Macau 999078, China; 3 UMacau Zhuhai Research Institute, Zhuhai 519031, China; 4 Department of Statistics, North Carolina State University, Raleigh, NC 27695, USA; hwei4@ncsu.edu; 5 Graduate School of Arts and Science, Yale University, New Haven, CT 06510-8034, USA; shouzheng.zhang@yale.edu

**Keywords:** density estimation, Elastic-net, measurement errors, support recovery, multi-mode data

## Abstract

This paper aims to estimate an unknown density of the data with measurement errors as a linear combination of functions from a dictionary. The main novelty is the proposal and investigation of the corrected sparse density estimator (CSDE). Inspired by the penalization approach, we propose the weighted Elastic-net penalized minimal $\ell_{2}$-distance method for sparse coefficients estimation, where the adaptive weights come from sharp concentration inequalities. The first-order conditions holding a high probability obtain the optimal weighted tuning parameters. Under local coherence or minimal eigenvalue assumptions, non-asymptotic oracle inequalities are derived. These theoretical results are transposed to obtain the support recovery with a high probability. Some numerical experiments for discrete and continuous distributions confirm the significant improvement obtained by our procedure when compared with other conventional approaches. Finally, the application is performed in a meteorology dataset. It shows that our method has potency and superiority in detecting multi-mode density shapes compared with other conventional approaches.

## 1. Introduction

Over the years, the mixture models have been extensively applied to model unknown distributional shapes in astronomy, biology, economics, and genomics (see [1] and references therein). The distributions of real data involving potential complex variables often show multi-mode and heterogeneity. Due to the flexibility, it also appears in various distribution-based statistical techniques, such as cluster analysis, discriminant analysis, survival analysis, and empirical Bayesian inference. Flexible mixture models can naturally represent how the data are generated as mathematical artifacts. Theoretical results show that the mixture can approximate any density in the Euclidean space well, and the amount of the mixture can also be finite (for example, a mixture of several Gaussian distributions). Although the mixture model is inherently attractive to the statistical modeling, it is well-known that it is difficult to infer (see [2,3]). From the computational aspect, the optimization problems of mixture models are non-convex. Although existing computational methods, such as EM and various MCMC algorithms, can make the mixture model fit the data relatively easily. It should be emphasized that the mixture problems are essentially challenging, even unrecognizable, and the number of components (says, the order selection) is hard to determine (see [4]). There is a large amount of literature on its approximation theory, and various methods have been proposed to estimate the components (see [5] and references therein).

The nonparametric method and combinatorial method in density estimation were well studied in [6,7], as well as [8]. These can consistently estimate the number of the mixture’s components when the components have known functional forms. When the number of candidate components is large, the non-parametric method becomes computationally infeasible. Fortunately, the high-dimensional inference would compensate for this gap and guarantee the corrected identification of the mixture components with a probability tending to one. With the advancement of technology, high-dimensional problems have been applied at the forefront of statistical research. The high-dimensional inference method has been applied to the infinite mixture models with a sparse mixture of p→∞ components, which is an interesting and challenging problem (see [9,10]). We propose an improvement of the sparse estimation strategy proposed in [9], in which Bunea et al. propose a $\ell_{1}$-type penalty [11] to obtain a sparse density estimate (SPADES). At the same time, we add a $\ell_{2}$-type penalty and extend the oracle-inequality results to our new estimator.

In the real data, we often encounter the situation that the i.i.d. samples $X_{i}=Z_{i}+\varepsilon_{i}$ are contained by some zero-mean measurement errors ${\left\{\varepsilon_{i}\right\}}_{i=1}^{n}$; see [12,13,14,15,16]. For density estimation of ${\left\{Z_{i}\right\}}_{i=1}^{n}$, if there exists orthogonal basis functions, the estimation method is quite easy. In the measurement errors setting, however, finding an orthogonal-based density function is not easy (see [17]). Ref. [17] suggests the assumption that the conditional distribution function of $X_{i}$ given $Z_{i}$ is known. This condition is somewhat strong since most conditional distributions cannot obtain the explicit formula (except the Gaussian distribution). To address this predicament, particularly with nonorthogonal base functions, the SPADES model is attractive and makes the situation easier to deal with. Based on the SPADES method, our approach is an Elastic-net calibration approach, which is simpler and more interpreted than the conditional inference procedure proposed by [17]. In this paper, we proposed the corrected loss function to debiasing the measurement errors, and this is motivated by [18]. The main problem of considering measurement errors in various statistical models is that they are responsible for the bias of the classical statistical estimates; this is true, e.g., in linear regression, which has traditionally been the main focus of studies related to measurement errors. Debiasing represents an important task in various statistical models. In linear regression, it can be performed in the basic measurement errors (ME) model, which is also denoted as the Errors-in-variables (EIV or EV) model, if it is possible to estimate the variability of measurement errors (see [19,20]). We derive the real variable selection consistency based on weighted $\ell_{1}$+ $\ell_{2}$ penalty [21]. At the same time, some theoretical results of SPADES only contain the situation of the equal weights setting, which is not plausible in the sense of adaptive (data-dependent) penalized estimation. Moreover, we perform the Poisson mixture model to approximate the complex discrete distribution in the simulation part, while existing papers only emphasize the performance of continuous distribution models. Note that the multivariate kernel density estimator can only deal with a continuous distribution, and it requires a multivariate bandwidths section, while our method is dimensional-free (the number of the required tuning parameters is only two). There has been quite a lot of work in this area, starting with [22].

There are several differences between our article and [9]. The first point is that the upper bound of non-asymptotic oracle inequality in in our Theorems 1 and 2 is tighter than Theorems 1 and 2 in [9], and the optimal weighted tuning parameters are derived. The second point is that the $\ell_{1}$-penalized techniques are applied in [9] to estimate the sparse density. Still, this paper considers the estimation of density functions in the presence of a classical measurement error. We opt to use an Elastic-net criterion function to estimate the density, which is taken to be approximated by a series of basis functions. The third point is that the tuning parameters are chosen by the coordinate descent algorithm in [9], and the mixture weights are calculated by the generalized bisection method (GBM). However, this paper directly calculates the optimal weights, so our algorithm is more accessible to implement than [9].

This paper is presented as follows. Section 2 introduces the density estimator, which can deal with measurement errors. This section introduces data-dependent weights for the Lasso penalty, and the weights are derived by the event of KKT conditions holding a high probability. In Section 3, we give a condition that can accurately estimate the weights of the mixture, with a probability tending to 1. We show that, in an increasing dimensional mixture model under the local coherence assumption, if the tuning parameter is higher than the noise level, the recovery of the mixture components can hold with a high probability. In Section 4, we study the performance of our approach on artificial data generated from mixture Gaussian or Poisson distributions compared with other conventional methods, which indeed shows the improvement by employing our procedures. Moreover, the simulation also demonstrates that our method is better than the traditional EM algorithm, even under a low-dimensional model. Considering the multi-modal density aspect of the meteorology dataset, our proposed estimator has a stronger ability to detect multiple modes for the underlying distribution compared with other methods, such as SPADES or un-weighted Elastic-net estimator. Section 5 is the summary, and the proof of theoretical results is delivered in the Appendix A.

## 2. Density Estimation

### 2.1. Mixture Models

Suppose that ${\left\{Z_{i}\right\}}_{i=1}^{n}\in R^{d}$ are independent random variables with a common unknown density *h*. However, the observations are contaminated with measurement errors ${\left\{\varepsilon_{i}\right\}}_{i=1}^{n}$ as latent variables, the observed data are actually $X_{i}=Z_{i}+\varepsilon_{i}$. Let ${\left\{h_{j}\right\}}_{j=1}^{W}$ be a series of density functions (such as Gaussian density or Poisson mass function), and ${\left\{h_{j}\right\}}_{j=1}^{W}$ are also called basis functions. Assume that the *h* belongs to the linear combination of ${\left\{h_{j}\right\}}_{j=1}^{W}$. The $W:=W_{n}$ is a function of *n*, which is particularly intriguing for us since there may be W≫n (the high-dimensional setting). Let $\beta^{*}:=\left(\beta_{1}^{*},\cdots ,\beta_{W}^{*}\right)\in R^{W}$ be the unknown true parameter. Assume that

- (H.1): the $h:=h_{\beta^{*}}$ is defined as
  (1) $$Z∼h\left(z\right):=h_{\beta^{*}}\left(z\right)=\sum_{j=1}^{W}\beta_{j}^{*}h_{j}\left(z\right),\;\;\mathrm{with}\;\sum_{j=1}^{W}\beta_{j}^{*}=1.$$

If the base is orthogonal and there are no measurement errors, a perfectly natural method is to estimate *h* by an orthogonal series of estimators in the form of $h_{\tilde{\beta }}$, where $\tilde{\beta }$ has the coordinates ${\tilde{\beta }}_{j}=\frac{1}{n}\sum_{i=1}^{n}h_{j}\left(X_{i}\right)$ (see [17]). However, this estimator depends on the choice of *W*, and a data-driven selection of *W* or the threshold needs to be adaptive. This estimator can only be applied to W≤n. Nevertheless, we want to solve more general problems for W>n, and the base functions ${\left\{h_{j}\right\}}_{j=1}^{W}$ may not orthogonal.

We aim to achieve the best convergence for the estimator when the *W* is not necessarily less than *n*. Theorem 33.2 in [5] states that any smooth density can be well-approximated by a finite mixture of some continuous functions. However, Theorem 33.2 in [5] does not confirm how many components *W* are required for the mixture. Thus, the hypothesis of the increasing-dimensional *W* is reasonable. For discrete distributions, there is also a similar mixture density approximation—see Remark of Theorem 33.2 in [5].

### 2.2. The Density Estimation with Measurement Errors

This subsection aims to construct a sparse estimator for the density $h\left(z\right):=h_{\beta^{*}}\left(z\right)$ as a linear combination of known densities.

Recall the definition of the $L_{2}\left(R^{d}\right)$ norm $∥f∥={\left(\int_{R^{d}}f^{2}\left(x\right)dx\right)}^{\frac{1}{2}}$. For $f,g\in L_{2}\left(R^{d}\right)$, let $<f,g>=\int_{R^{d}}f\left(x\right)g\left(x\right)dx$ be the inner product. If two functions *f* and *g* satisfy <f,g>=0, then we call these two functions are orthogonal. Note that if the density h(z) belongs to $L_{2}\left(R^{d}\right)$ and assume that ${\left\{X_{i}\right\}}_{i=1}^{n}$ has the same distribution *X*, for any $f\in L_{2}$, we have $<f,h>=\int_{R^{d}}f\left(x\right)h\left(x\right)dx=Ef\left(X\right)$. If h(x) is the density function for a discrete distribution, the integral is replaced by summation, and we can define the inner product as $<f,h>:=\sum_{k\in Z^{d}}f(k)h(k)$.

For true observations ${\left\{Z_{i}\right\}}_{i=1}^{n}$, we minimize the ${∥h_{\beta }-h∥}^{2}$ on $\beta \in R^{W}$ to obtain the estimate of $h\left(z\right):=h_{\beta^{*}}\left(z\right)$, i.e., minimizing

$$\begin{array}{c} {∥h_{\beta }-h∥}^{2}={∥h∥}^{2}+{∥h_{\beta }∥}^{2}-2<h_{\beta },h>={∥h∥}^{2}+{∥h_{\beta }∥}^{2}-2Eh_{\beta }\left(Z\right)\propto -2Eh_{\beta }\left(Z\right)+{∥h_{\beta }∥}^{2}, \end{array}$$

which implies that minimizing the ${∥h_{\beta }-h∥}^{2}$ is equivalent to minimizing

(2) $$\begin{array}{c} -2Eh_{\beta }\left(Z\right)+{∥h_{\beta }∥}^{2}\approx -\frac{2}{n}\sum_{i=1}^{n}h_{\beta }\left(Z_{i}\right)+{∥h_{\beta }∥}^{2}. \end{array}$$

It is plausible to assign more constrains for the candidate set of β in the optimization, for example, the $\ell_{1}$ constrains ${∥\beta ∥}_{1}\leq a$, where *a* is the tuning parameter. More adaptively, we prefer to use the weighted $\ell_{1}$ restriction $\sum_{j=1}^{W}\omega_{j}\left|\beta_{j}\right|\leq a$, where the weights $\omega_{j}$’s are data-dependent and will be specified later. From [23], we add Elastic-net penality $2\sum_{j=1}^{W}\omega_{j}\left|\beta_{j}\right|+c\sum_{j=1}^{W}\beta_{j}^{2}$ with tuning parameter *c*, which is in regards to the measurement errors (see [24,25]) for a similar purpose. We would have c=0 in the situation without measurement errors. The *c* indeed becomes larger if the measurement errors become more serious, i.e., we can say that the *c* is proportional to the increasing variability of the measurement errors. It is different from SPADES since adjusting for the measurement errors is important for accurately describing the relationship between the observed varables and the outcomes of interest.

From the discussion above, now we propose the following *Corrected Sparse Density Estimator* (CSDE):

(3) $$\begin{array}{c} \hat{\beta }:=\hat{\beta }\left(\omega_{1},\cdots ,\omega_{W}\right)=\underset{\beta \in R^{W}}{\arg\min}\left\{-\frac{2}{n}\sum_{i=1}^{n}h_{\beta }\left(X_{i}\right)+{∥h_{\beta }∥}^{2}+2\sum_{j=1}^{W}\omega_{j}\left|\beta_{j}\right|+c\sum_{j=1}^{W}\beta_{j}^{2}\right\} \end{array}$$

where the *c* is the tuning parameter for $\ell_{2}$-penalty, and the *c* also presents the correction for adjusting the measurement errors in our observations.

For CSDE, if ${\left\{h_{j}\right\}}_{j=1}^{W}$ is an orthogonal system, it can be clearly seen that the CSDE estimator is consistent with the soft thresholding estimator, and the explicit solution is ${\hat{\beta }}_{j}=\frac{{\left(1-\omega_{j}/|{\tilde{\beta }}_{j}|\right)}_{+}{\tilde{\beta }}_{j}}{1+c}$, where ${\tilde{\beta }}_{j}=\frac{1}{n}\sum_{i=1}^{n}h_{j}\left(X_{i}\right)$ and $x_{+}=\max\left(0,x\right)$. In this case, we can see that $\omega_{j}$ is the threshold of the *j*-th component of the simplest mean estimator $\tilde{\beta }=\left({\tilde{\beta }}_{1},\cdots ,{\tilde{\beta }}_{W}\right)$.

From the sub-differential of the convex optimization, the corresponding Karush–Kuhn–Tucker conditions (necessary and sufficient first-order condition) for the minimizer in Equation (3) is

**Lemma** **1** (KKT conditions, Lemma 4.2 of [26])**.** *Let* k∈{1,2,⋯,W} *and* c>0. *Then, a necessary and sufficient condition for CSDE to be a solution of Equation (3)*

1. ${\hat{\beta }}_{k}:\neq 0$*if*$\frac{1}{n}\sum_{i=1}^{n}h_{k}\left(X_{i}\right)-\sum_{j=1}^{W}{\hat{\beta }}_{j}<h_{j},h_{k}>-c{\hat{\beta }}_{k}=w_{k}\mathrm{sign}\left({\hat{\beta }}_{k}\right).$
2. ${\hat{\beta }}_{k}=0$*if*$\left|\frac{1}{n}\sum_{i=1}^{n}h_{k}\left(X_{i}\right)-\sum_{j=1}^{W}{\hat{\beta }}_{j}<h_{j},h_{k}>-c{\hat{\beta }}_{k}\right|\leq w_{k}.$

Since all values of $\beta_{j}^{*}$ are non-negative, when conducting minimization in Equation (3), we have to put a non-negative restriction for optimizing Equation (3).

Due to the computational feasibility and optimal first-order conditions, we prefer an adaptively weighted Lasso penalty as a convex adaptive $\ell_{1}$ penalization. We require that the larger weights are assigned to the coefficients of unimportant covariates, while significant covariates accompany the smaller weights. So, the weights represent the importance of the covariates. The larger (smaller) weights shrink to zero more easily (difficultly) than the unweighted Lasso, with appropriate or even optimal weights, leading to less bias and more efficient variable selection. The derivation of the weights will be given in Section 3.1.

In the end of this part, we will illustrate that in the mixture models, even without measurement errors, Equation (1) cannot be partially transformed into the linear model $Y=X^{T}\beta +\varepsilon$, where *Y* is the *n*-dimensional response variables, X is the W×n-dimensional fixed design matrix, β is a *W*-dimensional vector of model parameters, the ε is a n×1-dimensional vector for random error terms with zero mean and finite variance. Consider the least square objective function U(β) for estimating β, $U\left(\beta \right)={\left(Y-X^{T}\beta \right)}^{T}\left(Y-X^{T}\beta \right)=-2Y^{T}X^{T}\beta +\beta^{T}XX^{T}\beta +Y^{T}Y.$ Minimizing U(β) is equivalent to minimizing $U^{*}\left(\beta \right)$ in Formula (4)

(4) $$U^{*}\left(\beta \right)=-2Y^{T}X^{T}\beta +\beta^{T}XX^{T}\beta .$$

Comparing the objective function in Formula (4) with Equation (2), it is easy to obtain $Y={\left(\frac{1}{n},\frac{1}{n},\cdots ,\frac{1}{n}\right)}^{T},\;\beta ={\left(\beta_{1},\beta_{2},\cdots ,\beta_{W}\right)}^{T},\;X=\left(\begin{array}{ccc} h_{1}\left(X_{1}\right) & \cdots & h_{1}\left(X_{n}\right)\\ \vdots & \ddots & \vdots \\ h_{W}\left(X_{1}\right) & \cdots & h_{W}\left(X_{n}\right) \end{array}\right).$ Substituting *Y*, X and β into a linear regression model, we obtain

$${\left(\begin{array}{c} \frac{1}{n}\\ \vdots \\ \frac{1}{n} \end{array}\right)}_{n\times 1}={\left(\begin{array}{ccc} h_{1}\left(X_{1}\right) & \cdots & h_{W}\left(X_{1}\right)\\ \vdots & \ddots & \vdots \\ h_{1}\left(X_{n}\right) & \cdots & h_{W}\left(X_{n}\right) \end{array}\right)}_{n\times W}{\left(\begin{array}{c} \beta_{1}\\ \vdots \\ \beta_{W} \end{array}\right)}_{W\times 1}+{\left(\begin{array}{c} \varepsilon_{1}\\ \vdots \\ \varepsilon_{n} \end{array}\right)}_{n\times 1}.$$

Then,

(5) $$\varepsilon_{i}=\frac{1}{n}-\sum_{j=1}^{W}\beta_{j}h_{j}\left(X_{i}\right),\;i=1,2,\cdots ,n.$$

It can be seen from Equation (5) that the value of $\varepsilon_{i}$ is no longer random if X was the fixed design matrix. Furthermore, even for a random design X, take the expectation on both sides of Equation (5), and one can find that the left side is not equal to the right side, that is, $E\left(\varepsilon_{i}\right)=0=\frac{1}{n}-\sum_{j=1}^{W}\beta_{j}Eh_{j}\left(X_{i}\right).$ It leads to an additional requirement $\sum_{j=1}^{W}\beta_{j}Eh_{j}\left(X_{i}\right)=\frac{1}{n}\to 0$, which is meaningless as n→∞, since all $\beta_{j}$ and $h_{j}$ are positive. This is a contradiction to $\sum_{j=1}^{W}\beta_{j}Eh_{j}\left(X_{i}\right)>0$ for all *n*.

Both of the two situations above contradict the definition of the assumed linear regression model. Hence, we cannot convert the estimation of Equation (1) into the estimation problem of linear models. Thus, the existing oracle inequalities are not applicable anymore, and we will propose new ones later. However, we can transform the mixture models to a corrected score Dantzig selector, such as in [27]. Although [10] studies the oracle inequalities for adaptive Dantzig density estimation, their study does not contain the error-in-variables framework and the support recovery content.

## 3. Sparse Mixture Density Estimation

In this section, we will present the oracle inequalities for estimators $\hat{\beta }$ and $h_{\hat{\beta }}$. The core of this section consists of five main results corresponding to the oracle inequalities for estimated density (Theorems 1 and 2), upper bounds on $\ell_{1}$-estimation error (Corollaries 1 and 2) and support consistency (Theorem 3) as the byproduct of Corollary 2.

### 3.1. Data-Dependent Weights

The weights $\omega_{j}$’s are chosen adequately such that the KKT conditions for stochastic optimization problems have a high probability of being satisfied.

As mentioned before, the weights in Equation (3) rely on the observed data since we calculate the weights, ensuring the KKT conditions hold with a high probability. The weighted Lasso estimates could have less $\ell_{1}$ estimation error than Lasso estimates (see the simulation part and [28]). Next, we need to consider what kind of data-dependent weight configuration can enable the KKT conditions to be satisfied with a high probability. A way to obtain data-dependent weights is to apply a concentration inequality for a weighted sum of independent random variables. Moreover, the weights should be a known data function without any unknown parameters. A criterion can help obtain the weights grounded on McDiarmid’s inequality (see [29] for more details).

**Lemma** **2.** *Suppose $X_{1},\cdots ,X_{n}$ are independent random variables, and all values belong to a set A. Let $f:A^{n}\to R$ be a function and satisfy the bounded difference conditions*

$$\sup_{x_{1},\cdots ,x_{n},x_{s}^{\prime }\in A}\left|f\left(x_{1},\cdots ,x_{n}\right)-f\left(x_{1},\cdots ,x_{s-1},x_{s}^{\prime },x_{s+1},\cdots ,x_{n}\right)\right|\leq C_{s},$$

*then for all*t>0, $P\left\{|f\left(X_{1},\cdots ,X_{n}\right)-Ef\left(X_{1},\cdots ,X_{n}\right)|\geq t\right\}\leq 2\exp\left\{-\frac{2t^{2}}{\sum_{s=1}^{n}C_{s}^{2}}\right\}.$

We define the KKT conditions of optimization evaluated at $\beta^{*}$ (it is from the sub-gradient of the optimization function evaluated at $\beta^{*}$) by the events below:

$$F_{k}\left(\omega_{k}\right):=\left\{\left|\frac{1}{n}\sum_{i=1}^{n}h_{k}\left(X_{i}\right)-\sum_{j=1}^{W}\beta_{j}^{*}<h_{j},h_{k}>-c\beta_{k}^{*}\right|\leq \omega_{k}\right\},k=1,2,\cdots ,W.$$

Assume that

- (H.2): $\exists \;L_{k}>0$ s.t. ${∥h_{k}∥}_{\infty }=\max_{1\leq i\leq n}\left|h_{k}\left(X_{i}\right)\right|\leq 2L_{k}$;
- (H.3): $0<\max_{1\leq j\leq W}\left|\beta_{j}^{*}\right|\leq B$.

(H.2) is an assumption in sparse $\ell_{1}$ estimation, and the assumption (H.3) is a classical compact parameter space assumption in sparse high-dimensional regressions (see [9,25]).

Next, we check that the event $F_{k}\left(\omega_{k}\right)$ is hold with high probability. Note that $Eh_{k}\left(X_{i}\right)=\sum_{j=1}^{W}\beta_{j}^{*}<h_{j},h_{k}>$ (which is free of $X_{i}$), we find

$$\begin{array}{c} \frac{1}{n}\left|\sum_{i=1}^{n}h_{k}\left(X_{i}\right)-\left(\sum_{i\neq s}^{n}h_{k}\left(X_{i}\right)+h_{k}\left(X_{s}^{\prime }\right)\right)\right|=\frac{1}{n}\left|h_{k}\left(X_{s}\right)-h_{k}\left(X_{s}^{\prime }\right)\right|\leq \frac{1}{n}\left(\right|h_{k}\left(X_{s}\right)\left|+\right|h_{k}\left(X_{s}^{\prime }\right)\left|\right)\leq \frac{4L_{k}}{n}, \end{array}$$

where the last inequality is due to $|h_{k}\left(X_{i}\right)|\leq \max_{1\leq i\leq n}\left|h_{k}\left(X_{i}\right)\right|\leq 2L_{k}$.

Next, we apply the McDiarmid’s inequality on the event $F_{k}^{c}\left(\omega_{k}\right)$ by (H.3). Then

$$\begin{array}{cc} P(F_{k}^{c}\left(\omega_{k}\right)) & =P\left\{\left|\frac{1}{n}\sum_{i=1}^{n}h_{k}\left(X_{i}\right)-\sum_{j=1}^{W}\beta_{j}^{*}<h_{j},h_{k}>-c\beta_{k}^{*}\right|\geq \omega_{k}\right\}\\ (\mathrm{by}\;\left(H.3\right))\; & \leq P\left\{\left|\frac{1}{n}\sum_{i=1}^{n}h_{k}\left(X_{i}\right)-Eh_{k}\left(X_{i}\right)\right|\geq \omega_{k}-cB\right\}\\ (\mathrm{define}\;{\tilde{\omega }}_{k}:=\omega_{k}-cB>0)\; & \leq 2\exp\left\{-\frac{2{\tilde{\omega }}_{k}^{2}}{16L_{k}^{2}/n}\right\}=2\exp\left\{-\frac{n{\tilde{\omega }}_{k}^{2}}{8L_{k}^{2}}\right\}=:\frac{\delta }{W},\;0<\delta <1. \end{array}$$

Considering the previous line,

(6) $$\omega_{k}:=2\sqrt{2}L_{k}\sqrt{\frac{1}{n}\log\frac{2W}{\delta }}+cB=:2\sqrt{2}L_{k}v\left(\delta /2\right)+cB,\;\mathrm{where}\;v=v\left(\delta \right):=\sqrt{\frac{1}{n}\log\frac{W}{\delta }}.$$

The weight $\omega_{k}$ in our paper is different from [9], which gives the un-shift version ($\overset{ˇ}{\omega_{k}}=4L_{k}\sqrt{\frac{1}{n}\log\frac{W}{\delta /2}}$), due to the Elastic-net penalty. Define the modified KKT conditions:

(7) $$K_{k}\left(\omega_{k}\right):=\left\{\right|\frac{1}{n}\sum_{i=1}^{n}h_{k}\left(X_{i}\right)-\sum_{j=1}^{W}\beta_{j}^{*}<h_{j},h_{k}>\left|\leq {\tilde{\omega }}_{k}\right\},k=1,2,\cdots ,W$$

which hold with probability of at least $1-2\exp\left\{-\frac{n{\tilde{\omega }}_{k}^{2}}{8L_{k}^{2}}\right\}$.

### 3.2. Non-Asymptotic Oracle Inequalities

Introduced by [30], oracle inequality is a powerful non-asymptotic and analytical tool that seeks to provide the distance between the obtained estimator and a true estimator. The sharp oracle inequality connects the optimal convergence of an obtained estimator compared with the true parameter (see [31,32]).

For $\forall \beta \in R^{W}$, let $I\left(\beta \right)=\{j\in \left\{1,\cdots ,W\right\}:\beta_{j}\neq 0\}$ be the indices corresponding to the non-zero components of the vector β, i.e., the support in mathematical jargon. If there is no ambiguity, we would like to write $I(\beta^{*})$ as $I_{*}$ for simplicity. Define $W\left(\beta \right)=\sum_{j=1}^{W}I\left(\beta_{j}\neq 0\right)$ as the number of its non-zero components, where I(·) represents the indicative function. Let $\sigma_{j}^{2}=Var\left(h_{j}\left(X_{1}\right)\right),\;1\leq j\leq W$.

Below, we will state the non-asymptotic oracle inequalities for $h_{\hat{\beta }}$ (with the probability at least 1−δ(W,n) for any integer *W* and *n*), which measures the $L_{2}$ distance between $h_{\hat{\beta }}$ and *h*. For $\beta \in R^{W}$, define the correlation for the two base densities: $h_{i}$ and $h_{j}$, $\rho_{W}\left(i,j\right)=\frac{<h_{i},h_{j}>}{∥h_{i}∥∥h_{j}∥}$, i,j=1,⋯,W. Our results will be established under the local coherence condition, and we define the maximal local coherence as:

$$\rho \left(\beta \right)=\max_{i\in I(\beta )}\max_{j\neq i}\left|\rho_{W}\left(i,j\right)\right|.$$

It is easy to see that ρ(β) measures the separation of the variables in the set I(β) from one another and the rest. The degree of separation is measured in terms of the size of the correlation coefficients. However, the regular condition introduced by this coherence may be too strong. It may exclude cases that the “correlation” can be relatively significant for a small number of pairs (i,j) and almost zero otherwise. Thus, we consider the definition of the cumulative local coherence given by [9]: $\rho_{*}\left(\beta \right)=\sum_{i\in I(\beta )}\sum_{j>i}\left|\rho_{W}\left(i,j\right)\right|$. Define $H\left(\beta \right)=\max_{j\in I(\beta )}\frac{\omega_{j}}{v\left(\delta /2\right)∥h_{j}∥},\;F=\max_{1\leq j\leq W}\frac{v\left(\delta /2\right)∥h_{j}∥}{{\tilde{\omega }}_{j}}=\max_{1\leq j\leq W}\frac{∥h_{j}∥}{2\sqrt{2}L_{j}},$ where ${\tilde{\omega }}_{j}:=2\sqrt{2}L_{j}v\left(\delta /2\right)$.

By using the definition of $\rho_{*}\left(\beta \right)$ and the notations above, we present the main results of this paper, which lays the foundation for the oracle inequality of the estimated mixture coefficients.

**Theorem** **1.** *Under (H.1)–(H.3), let $c=\frac{\min_{1\leq j\leq W}\left\{{\tilde{\omega }}_{j}\right\}}{B}$ and a given constant 0<γ≤1. If the true base functions ${\left\{h_{j}\right\}}_{j=1}^{W}$ conform to the cumulative local coherence assumption for all $\beta \in R^{W}$,*

(8) $$12FH\left(\beta \right)\rho_{*}\left(\beta \right)\sqrt{W(\beta )}\leq \gamma ,$$

*then the $\hat{\beta }$ of the optimization problem in Equation (3) has the following oracle inequality with a probability at least 1−δ,*

$$\begin{array}{cc} & {∥h_{\hat{\beta }}-h∥}^{2}+\frac{\alpha_{opt1}\left(1-\gamma \right)}{\left(\alpha_{opt1}-1\right)}\sum_{j=1}^{W}{\tilde{\omega }}_{j}\left|{\hat{\beta }}_{j}-\beta_{j}\right|+\frac{\alpha_{opt1}}{\alpha_{opt1}-1}\sum_{j=1}^{W}c{\left({\hat{\beta }}_{j}-\beta_{j}\right)}^{2}\\ \leq & \frac{\alpha_{opt1}+1}{\alpha_{opt1}-1}{∥h_{\beta }-h∥}^{2}+\frac{18\alpha_{opt1}^{2}}{\alpha_{opt1}-1}H^{2}\left(\beta \right)v^{2}\left(\delta /2\right)W\left(\beta \right), \end{array}$$

*where $\alpha_{opt1}=1+\sqrt{1+\frac{{∥h_{\beta }-h∥}^{2}}{9H^{2}\left(\beta \right)v^{2}\left(\delta /2\right)W\left(\beta \right)}}$.*

It is worthy to note that here we use $\sqrt{W(\beta )}$ instead of W(β), and the latter is used in [9]. The upper bound of the oracle inequality by Theorem 1 is sharper than the upper bound of Theorem 1 in [9]. Further, we give the value of the optimal $\alpha_{opt1}$, but [9] did not give it. The reason for this phenomenon is quite clean actually: from the proof, it is due to ineuqality (A5). Now, let us address the sparse Gram matrix $\Psi_{W}={\left(<h_{i},h_{j}>\right)}_{1\leq i,j\leq W}$ with a small number of non-zero elements in off-diagonal positions, define $\psi_{W}\left(i,j\right)$ as the element (i,j)-th of position $\psi_{W}$. Condition (8) in Theorem 1 can be transformed to the condition

$$12SH\left(\beta \right)\sqrt{W(\beta )}\leq \gamma ,$$

where the number *S* is called the sparse index of matrix $\Psi_{W}$, which is defined as $S=|\{\left(i,j\right):i,j\in \left\{1,\cdots ,W\right\},i>j\;\mathrm{and}\;\psi_{W}\left(i,j\right)\neq 0\}|$, where |A| is the number of elements of set *A*.

Sometimes the assumption in Condition (8) does not imply the positive definiteness of $\Psi_{W}$. Next, we give a similar oracle inequality that is valid under the hypothesis that the Gram matrix $\Psi_{W}$ is positive definite.

**Theorem** **2.** *Under the assumption of (H.1)–(H.3) and that the Gram matrix $\Psi_{W}$ is positive definite with a minimum eigenvalue greater than or equal to $\lambda_{W}>0$. For all $\beta \in R^{W}$, the $\hat{\beta }$ of the optimization problem in Equation (3) has the following oracle inequality with probability at least 1−δ,*

$$\begin{array}{cc} & {||h_{\hat{\beta }}-h∥}^{2}+\frac{\alpha_{opt2}}{\alpha_{opt2}-1}\sum_{j=1}^{W}{\tilde{\omega }}_{j}\left|{\hat{\beta }}_{j}-\beta_{j}\right|+\frac{\alpha_{opt2}}{\alpha_{opt2}-1}\sum_{j=1}^{W}c{\left({\hat{\beta }}_{j}-\beta_{j}\right)}^{2}\\ \leq & \frac{\alpha_{opt2}+1}{\alpha_{opt2}-1}{∥h_{\beta }-h∥}^{2}+\frac{576\alpha_{opt2}^{2}}{\alpha_{opt2}-1}\frac{G}{\lambda_{W}}v^{2}\left(\delta /2\right), \end{array}$$

*where*$G=G\left(\beta \right):=\sum_{j\in I(\beta )}L_{j}^{2}$*and*$\alpha_{opt2}=1+\sqrt{1+\frac{{∥h_{\beta }-h∥}^{2}}{288\frac{G}{\lambda_{W}}v^{2}\left(\delta /2\right)}}$.

**Remark** **1.** *The argument and result of Theorem 1 in this paper is more refined than the conclusion of Theorem 1 in [9] for Lasso by letting γ=1/2 and c=0. In addition, Theorems 1 and 2 of this paper, respectively, give the optimal α value of the density estimation oracle inequalities, namely $\alpha_{opt1}$, $\alpha_{opt2}$. It provides a potentially sharper bound for the $\ell_{1}$-estimation error bound.*

*Next, we will present the $\ell_{1}$-estimation error for the estimator $\hat{\beta }$ by Equation (3), and the weights are defined by Equation (6). For the technical point, we consider that $∥h_{j}∥=1$ for all j in Equation (3), i.e., the base functions are normalized. This normalization mimics the covariates’ standardization procedure when doing penalized estimations in generalized linear models. For simplicity, we put $L:=\max_{1\leq j\leq W}L_{j}$.*

For any other choice of v(δ/2) greater than or equal to $\sqrt{\frac{1}{n}\log\frac{2W}{\delta }}$, the conclusions of Section 3 are valid with a high probability. It imposes a restriction on the predictive performance of CSDE. As pointed out in [33], for the $\ell_{1}$-penalty in the regression, the adjusted sequence $\omega_{j}$ required for the corrected selection is usually larger than the adjusted sequence $\omega_{j}$ that produces a good prediction. The selection of the mixture density shown below is also true. Specifically, we will take the value $\beta =\beta^{*}$ and $v=v\left(\delta /2W\right)=\sqrt{\frac{\log(2W^{2}/\delta )}{n}}$, then $\alpha_{opt1},\alpha_{opt2}=2$. Below, we give the Corollaries of Theorems 1 and 2.

**Corollary** **1.** *Given the same conditions as Theorem 1 with $∥h_{j}∥=1$ for all j, let $\alpha_{opt1}=2$, then we have the following $\ell_{1}$-estimation error oracle inequality:*

(9) $$\begin{array}{c} \sum_{j=1}^{W}\left|{\hat{\beta }}_{j}-\beta_{j}^{*}\right|\leq \frac{72\sqrt{2}v\left(\delta /2W\right)W\left(\beta^{*}\right)}{1-\gamma }\frac{{\left(L+L_{\min}\right)}^{2}}{L_{\min}} \end{array}$$

*with probability at least 1−δ/W, where $L_{\min}=\min_{1\leq j\leq W}L_{j}$.*

**Corollary** **2.** *Given the same conditions as Theorem 2 with $∥h_{j}∥=1$ for all j, let $\alpha_{opt2}=2$, then we have the following oracle inequality, with probability at least 1−δ/W,*

$$\sum_{j=1}^{W}\left|{\hat{\beta }}_{j}-\beta_{j}^{*}\right|\leq \frac{288\sqrt{2}v\left(\delta /2W\right)G^{*}}{L_{\min}\lambda_{W}},\;where\;G^{*}=\sum_{j\in I_{*}}L_{j}^{2}.$$

If the number $W(\beta^{*})$ of the mixture indicator elements is much smaller than $\sqrt{n}$, then inequality (9) guarantees that the estimated $\hat{\beta }$ is close to the true $\beta^{*}$, and the $\ell_{1}$-estimation error will be presented in the numerical simulation in Section 4. Our results of Corollaries 1 and 2 are non-asymptotic for any *W* and *n*. The oracle inequalities are guiders for us to find an optimal tuning parameter with order $O(\sqrt{\frac{\log W}{n}})$ for a sharper estimation error and better prediction performance. This is also an intermediate and crucial result, which leads to the main results of correctly identifying the mixture components in Section 3.3. In the following section, we turn to cope with the identification of $I_{*}$. Corrected components are selected by the proposed oracle inequality for the weighted $\ell_{1}$+ $\ell_{2}$ penalty.

### 3.3. Corrected Support Identification of Mixture Models

In this section, we will study the results of the support recovery of our CSDE estimator. There are few results on support recovery, while most of the results are the consistency of the $\ell_{1}$-error and prediction errors. Here, we borrow the framework of [25,33]. They give many proof techniques to deal with the corrected support identification in linear models by $\ell_{1}+\ell_{2}$ regularization. Let $\hat{I}$ be the set of indicators consisting of non-zero elements of $\hat{\beta }$ in the given Equation (3). In other words, $\hat{I}$ is an estimate of the true support set $I\left(\beta^{*}\right):=I_{*}$. We will study $P(\hat{I}=I\left(\beta^{*}\right))\geq 1-\varepsilon$ for a given 0<ε<1 under some mild conditions.

To identify the $I_{*}$ consistently, we need more assumptions about some special correlation conditions than $\ell_{1}$-error consistency.

**Condition (A)**: $\rho_{*}\left(\beta^{*}\right)\leq \frac{LL_{\min}\lambda_{W}}{288G^{*}}.$

Moreover, we need an additional condition that the minimal signal should be higher than a threshold level and quantified by order of tuning parameter. Therefore, we state it as follows:

**Condition (B)**: $\min_{j\in I^{*}}\left|\beta_{j}^{*}\right|\geq 4\sqrt{2}v\left(\frac{\delta }{2W}\right)L$, where $v\left(\frac{\delta }{2W}\right):=\sqrt{\frac{1}{n}\log\frac{2W^{2}}{\delta }}$.

When performing simulation, Condition (B) is the theoretical guarantee that the minor magnitude of $\beta_{j}$ must be greater than a threshold value as a minimal signal condition. It is also called the Beta-min condition (see [26]).

**Theorem** **3.** *Let $0<\delta <\frac{1}{2}$ and define $\epsilon_{k}:=\left|E\left[h_{k}\left(X_{1}\right)\right]-E\left[h_{k}\left(Z_{1}\right)\right]\right|$. Assume that both conditions (A) and (B) are true and give the same conditions as Corollary 2, then*

$$P\left(\hat{I}=I_{*}\right)\geq 1-\left(4W{\left(\frac{\delta }{2W^{2}}\right)}^{{\left(1-\epsilon_{k}^{*}\right)}^{2}}+2\delta \right),\;where\;\epsilon_{k}^{*}=\epsilon_{k}/\sqrt{2}v\left(\delta /2W\right)L.$$

Under the Beta-min condition, the support estimation is very close to the true support of $\beta_{j}^{*}$. The probability of the event $\left\{\hat{I}=I_{*}\right\}$ is high when *W* is growing. The $\hat{\beta }$ recovers the corrected support with probability at least $1-(4W{\left(\frac{\delta }{2W^{2}}\right)}^{{\left(1-\epsilon_{k}^{*}\right)}^{2}}+2\delta )$. The result is non-asymptotic and it is true for any fixed *W* and *n*. There is a similar conclusion about support consistency (see Theorem 6 of [25]).

## 4. Simulation and Real Data Analysis

Ref. [9] proposes the SPADES estimation to deal with the samples for sparse mixture density, and they also derive an algorithm from complementing their theoretical result. Their findings successfully handle the high-dimensional adaptive density estimation to some degree. However, their algorithm is costly and unstable. In this section, we deal with the tuning parameter directly and compare our CSDE method with the SPADES method in [9] and other similar methods. In all cases here, we fix n=100 for W=81,131,211,321, which is known as the dimension of the unknown parameter $\beta^{*}$. The performance of each estimator is evaluated by the $\ell_{1}$-estimation error and the total variation (TV) distance between the estimator and the true value of $\beta^{*}$. The total variation (TV) error is defined as: $\mathrm{TV}\left(h_{\beta^{*}},h_{\hat{\beta }}\right)=\int \left|h_{\beta^{*}}\left(x\right)-h_{\hat{\beta }}\left(x\right)\right|\;dx.$

### 4.1. Tuning Parameter Selection

In [9], the $\lambda_{1}$ is chosen by the coordinate descent method, while the mixture weights are detected by GBM. However, in our article, the optimal weights can be computed directly. Thus, it is much easier to carry out than [9]. The $\ell_{1}$-penalty term $\sum_{j=1}^{W}\omega_{j}\left|\beta_{j}\right|$ with optimal weights are defined by $\omega_{k}:=2\sqrt{2}L_{k}v\left(\delta /2\right)+cB$, where $L_{j}={∥h_{j}∥}_{\infty }$, which usually can be computed easily for a continuous $h_{j}$.

For a discrete base density ${\left\{h_{j}\right\}}_{j=1}^{W}$, it can be estimated as the following approximation by using concentration inequalities from Exercise 4.3.3 of [34]: $\left|\mathrm{med}\left(X\right)-E\left(X\right)\right|\leq \sqrt{2Var(X)},\;\;\bar{x}\approx x_{med}\left(1+O(n^{-1})\right)\approx h^{-1}\left(L_{j}\right)\left(1+O(n^{-1})\right)$, where $\bar{x}$ and $x_{med}$ represent the sample mean and sample median, respectively, in each simulation, then we only need to select the $\lambda_{1}$ and $c=\lambda_{2}$, and they can be detected by the nesting coordinate descent method. Moreover, the precision level is assigned as ξ=0.001 in our simulation.

### 4.2. Multi-Modal Distributions

First, we examine our method in a multi-modal Gaussian model that is similar to the first model in [9]. However, our mixture Gaussian has a different variance, which leads the meaningful weights to our estimation. The density function for the i.i.d. sample $Z_{1},\ldots ,Z_{n}$ is assigned as follows:

(10) $$h_{\beta }^{*}\left(x\right)=\sum_{j=1}^{W}\beta_{j}^{*}\phi \left(x|aj,\sigma_{j}\right),$$

where $\phi \left(x|aj,\sigma_{j}\right)$ is the density of $N(aj,\sigma_{j}^{2})$. However, to estimate $\beta^{*}$, we only observe i.i.d. data $X_{1},\ldots ,X_{n}$ with density $g_{\beta_{j}^{*}}\left(x\right)=\sum_{j=1}^{W}\beta_{j}^{*}\phi \left(x|aj,\sqrt{1.1}\sigma_{j}\right)$. Put a=0.5,n=100 and

(11) $$\beta^{*}={\left(0_{8}^{T},0.2,0_{10}^{T},0.1,0_{5}^{T},0.1,0_{10}^{T},0.1,0_{10}^{T},0.1,0_{5}^{T},0.15,0_{10}^{T},0.15,0_{10}^{T},0.1,0_{W-76}^{T}\right)}^{T},$$

with $\sigma ={\left(1_{20}^{T},0.8_{6}^{T},0.6_{11}^{T},0.4_{11}^{T},0.6_{6}^{T},0.8_{11}^{T},1.2_{W-76}^{T}\right)}^{T}$.

We replicate the simulation N=100 times. Simulation results are presented in Table 1, from which we can see our method has more and more excellent performances as the *W* increases, which matches the non-asymptotic results in the previous section. The best performance is far better than the other three methods when W=321. It is worthy to note that the better approximation follows the increase in *W*, matching Equation (7) and Theorem 3 in our previous section.

**Figure 1:**
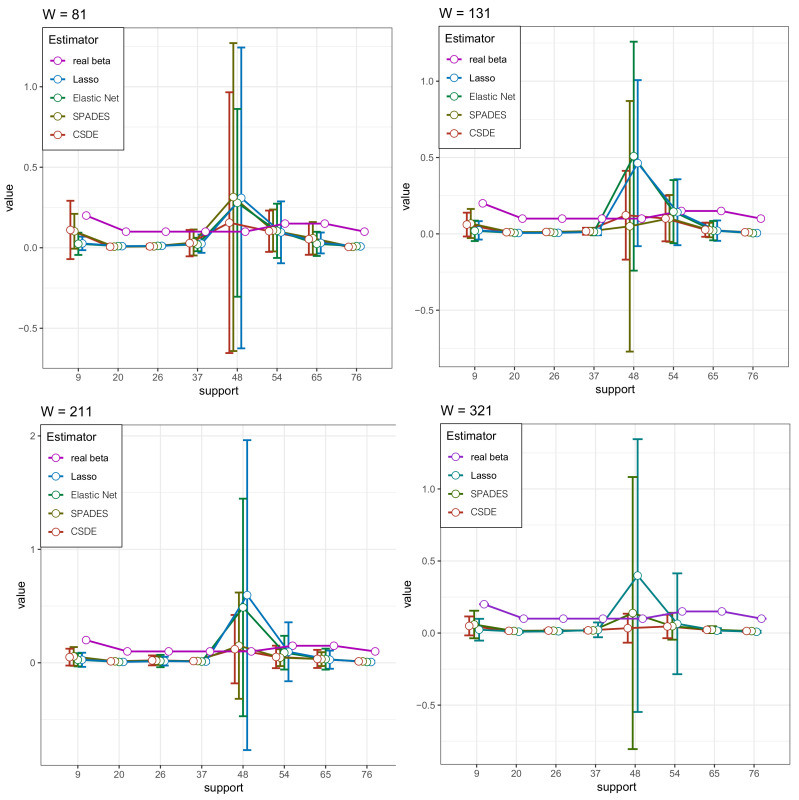
The simulation result in Section 4.2. The estimated support of $\beta^{*}$ by the four types of estimators, and the *W* is varying. The circles represent the means of the estimators under the four specific approaches, while the half vertical lines mean the standard deviations.

**Figure 2:**
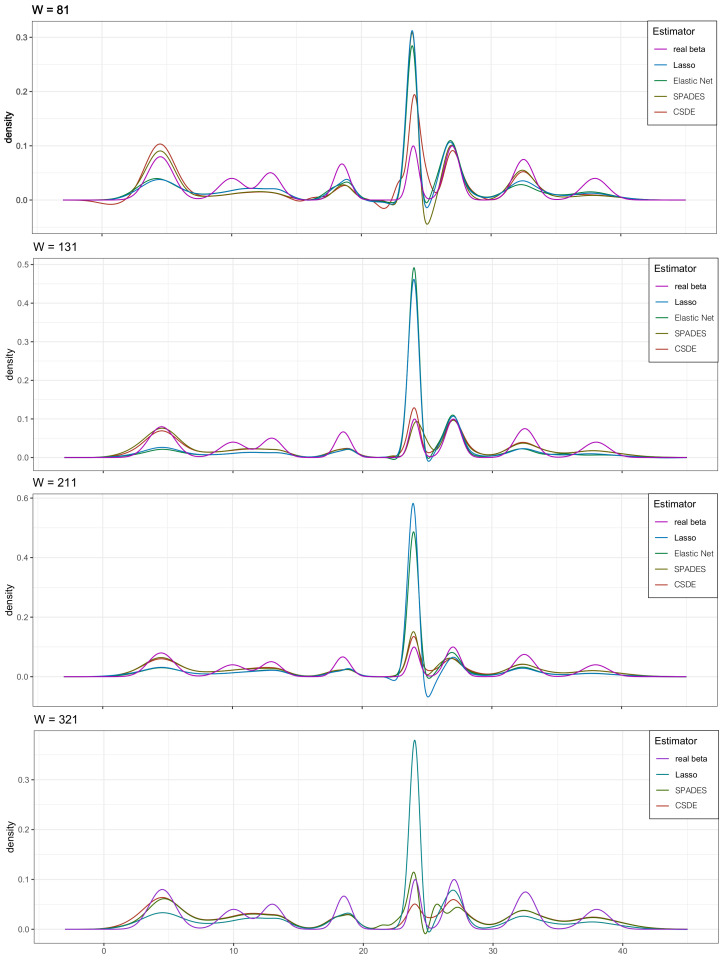
The simulation results in Section 4.2. The density map of the four estimators. The result of Elastic-net in W=321 is not be shown due to its poor performance.

We plot the solution path to compare the performance of the four estimators in $\beta_{j}\in I\left(\beta \right)$ for every *W* in Figure 1 (the result of Elastic-net in W=321 is not be shown due to its poor performance.). These figures also provide strong support for the above analysis. Meanwhile, we plot the probability densities of the several estimators and the true density to complement the visual sensory of the advantage in our method in Figure 2. The robust competency of detecting the multi-mode is shown (whereas other methods only find the most strongest signal, ignoring other meaningful but relatively weak signals).

### 4.3. Mixture of Poisson Distributions

We study the mixture of discrete distribution: the mixture Poisson distribution

(12) $$h_{\beta^{*}}\left(x\right)=\sum_{j=1}^{W}\beta_{j}^{*}p\left(x|\lambda_{j}=a\cdot j\right),$$

where $p\left(x|\lambda_{j}=a\cdot j\right)$ is the probability mass function (p.m.f.) of the Poisson distribution with mean $\lambda_{j}$. We set a=0.1 and

(13) $$\beta^{*}={\left(0_{8}^{T},0.2,0_{10}^{T},0.1,0_{5}^{T},0.1,0_{10}^{T},0.1,0_{10}^{T},0.1,0_{5}^{T},0.15,0_{10}^{T},0.15,0_{10}^{T},0.1,0_{W-75}^{T}\right)}^{T}.$$

The adjusted weights are calculated by Equation (3), and in discrete distributions, we define $\langle f,g\rangle =\sum_{k=1}^{\infty }f(k)g(k)$. Meanwhile, the Poisson random variable with measurement errors can be treated as a negative binomial random variable. Let $n(x|\lambda_{j},r)$ be the p.m.f. of the Poisson distribution with the mean $\lambda_{j}$ and dispersion parameter *r*. Suppose the observed data with sample size n=100 has the p.m.f.

(14) $$g_{\beta^{*}}\left(x\right)=\sum_{j=1}^{W}\beta_{j}^{*}n\left(x|\lambda_{j}=a\cdot j,r\right),$$

where r=6, which leads to an increment of variance from Poisson to the negative binomial distribution. Similarly, we replicate each simulation to estimate the parameter N=100 times with the sample from the mixture negative binomial distribution above. The result is shown in Table 2. The result is actually akin to that in the previous mixture Gaussian distribution, while the strong performance of our method is shown clearly when *W* is considerable.

### 4.4. Low-Dimensional Mixture Model

Surprisingly, our method has more competitive efficacy than some popular methods (such as EM algorithm), even the dimension *W* is relatively small. To see this, we introduce the following numerical experiments to estimate the weights of the low-dimensional Gaussian mixture model: the samples $X_{1},\cdots ,X_{n}$ come from the model: $h_{\beta^{*}}\left(x\right)=\sum_{j=1}^{W}\beta_{j}^{*}\phi \left(x|\mu_{j},\sigma_{j}\right).$ The updated equation for the EM algorithm in *t*-th step is:

$$\omega_{ij}^{\left(t\right)}=\frac{p_{j}^{\left(t\right)}\phi \left(x_{i};\mu_{t},\sigma_{t}\right)}{\sum_{s=1}^{W}p_{s}^{\left(t\right)}\phi \left(x_{i};\mu_{s},\sigma_{s}\right)},\;\beta_{j}^{\left(t+1\right)}=\frac{\sum_{i=1}^{W}\omega_{ij}^{\left(t\right)}}{\sum_{i=1}^{n}\sum_{j=1}^{W}\omega_{ij}^{\left(t\right)}}.$$

Here, we consider two scenarios:

$$\begin{array}{cc} \; & \;\left(1\right)\;W=6,\beta ={\left(0.3,0,0,0.3,0,0.4\right)}^{T},\mu ={\left(0,10,20,30,40,50\right)}^{T},\sigma ={\left(1,2,3,4,5,6\right)}^{T};\\ \; & \;\left(2\right)\;W=7,\beta ={\left(0.1,0,0,0.8,0,0,0.1\right)}^{T},\mu ={\left(0,1,2,3,4,5,6\right)}^{T},\\ & \;\;\;\;\;\;\;\sigma ={\left(0.3,0.2,0.2,0.1,0.2,0.2,0.3\right)}^{T}. \end{array}$$

For each scenario n=50, and the fitter levels (cessation level) in the EM approach and our method are both $\xi =10^{-4}$. A well-advised initial value in the EM approach is the equal weight.

We replicate the simulation N=100 times, and the optimal tuning parameters stem from the cross-validation (CV). Thus, under each simulation, they are not the same, albeit they are very close to each other. The result can be seen in Table 3.

### 4.5. Real Data Examples

Practically, we consider using our method to estimate some densities in the environmental science field. Wind, which is mercurial, has been an advisable object to study for a long time in meteorology. Please note that the wind’s speed at one specific location may not be diverse so we will use the wind’s azimuth angle with a more sparse density at two sites in China. Many types of research about the estimated density for wind exist, so there is a possibility of using our approach to cope with some difficulties in meteorology science.

**Figure 3:**
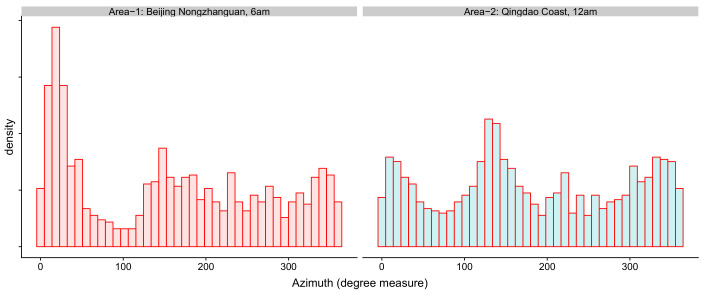
The sample histogram of the azimuth in Beijing Nongzhanguan at 6am and Qingdao Coast at 12 am.

There have been some very credible meteorological dataset. We used the ERA5 hourly data in [35] to continue our analysis. We want chose a continental area and a coastal area in China, so we chose Beijing Nongzhanguan and Qingdao Coast. The locations of these two areas are: (116.3125${}^{{}^{\circ}}$ E, 116.4375${}^{{}^{\circ}}$ E) × (39.8125${}^{{}^{\circ}}$ N, 39.8125${}^{{}^{\circ}}$ N). Take notice that the wind in one day may be highly correlated. Therefore, using the data at a specific time point of each day in a consecutive period as i.i.d. samples is reasonable. The sample histograms at 6 am in Beijing Nongzhanguan and at midnight on the Qingdao Coast are shown in Figure 3. Here, we used the data from 1 January 2013 to 12 December 2015.

As we can see, their density does multi-peak (we used 1095 samples). Now, we can use our approach to estimate the multi-mode densities based on a relatively small size of samples, which is only a tiny part of the whole data from 1 January 2013 to 12 December 2015. Because one year has about 360 days, we may assume that every day is a latent factor that forms the base density. Thus, the model is designed as $h_{\beta^{*}}\left(x\right)=\sum_{j=1}^{360}\beta_{j}^{*}\phi \left(x|\mu_{j},\sigma_{j}\right)$ with the mean and variance parameters $\mu ={\left(1,2,\ldots ,360\right)}^{T}$, $\sigma =t\cdot 1_{360}^{T}$, where *t* is the bandwidth (or tuning parameter). With the different sub-samples, the computed values are different.

Another critical issue is how to choose the tuning parameters $\lambda_{1}$ and $\lambda_{2}$. Then, we apply the cross-validation criterion, namely choosing $\lambda_{i}$, to minimize the difference between the two estimators derived from the separated samples in a random dichotomy.

**Figure 4:**
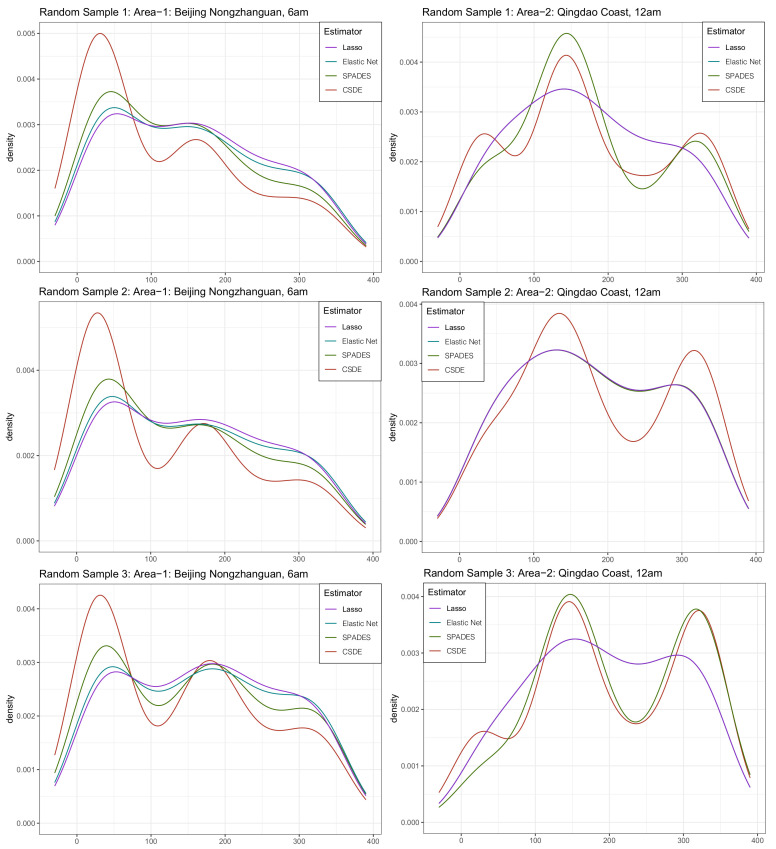
The density map of the four estimators’ approaches for the three random sub-samples from the real-world data in Section 4.5.

Now, start to construct the samples for the estimating procedure. Assume that an observatory wants to figure out some information about the two areas’ wind. However, it does not have intact data due to the limited budget at its inception. The only samples it has are several days’ information each month for the two areas, and these days scatter randomly. Furthermore, sample size n=168 exactly. These imperfect data increase the challenge of estimating a trustworthy density. We compared our method with other previous methods, in which appraising the difference between the complete data sample histogram and the estimated density under each method is for the evaluation. Notice that the samples are only a tiny part of the data, so the n=168<M(=360) is relatively small. The small sample and large dimension setting coincide with the non-asymptotic theory provided in the previous section. The estimated density has been shown in Figure 4.

In this practical application, our method vindicates its more efficient estimating performance and stability from its propinquity of the complete sample histogram, namely the productive capacity of detecting the shape of the multi-mode density and the stronger inclination to bear a resemblance to each other sub-sample (although some subtle nuances do exist because of the different sub-sample). An alternative approach can be to consider principles and tools of circular statistics, which has been reviewed in [36].

## 5. Summary and Discussions

The paper deals with the deconvolution problem using Lasso-type methods: the observations $X_{1},\cdots ,X_{n}$ are independent and generated from $X_{i}=Z_{i}+\varepsilon_{i}$, and the goal is to estimate the unknown density *h* of the $Z_{i}$. We assume that the function *h* can be written as $h\left(\cdot \right)=h_{\beta^{*}}\left(\cdot \right)=\sum_{j=1}^{W}\beta_{j}^{*}h_{j}\left(\cdot \right)$ based on some functions ${\left\{h_{j}\right\}}_{j=1}^{W}$ from a specific dictionary and propose estimating the coefficients of this decomposition with the Elastic-net method. For this estimator, we show that under some classical assumptions of the model, such as coherence of the Gram matrix, finite sample bounds for the estimation and the prediction errors valid with a relatively high probability can be obtained. Moreover, we prove a variable selection consistency result under a beta-min condition and conduct an extensive numerical study. The following estimation problem is also similar to the CSDE.

For future study, it is also interesting and meaningful to do hypothesis testing about the coefficients $\beta^{*}\in R^{W}$ in sparse mixture models. For a general function $h:R^{W}\mapsto R^{m}$ and a nonempty closed set $\Omega \in R^{m}$, we can consider

$$H_{0}:h\left(\beta^{*}\right)\in \Omega \;\mathrm{vs}.\;H_{1}:h\left(\beta^{*}\right)\notin \Omega .$$

It is possible to use [37] as a general approach to hypothesis testing within models with measurement errors.

## Figures and Tables

**Table 1:** The simulation results in Section 4.2. The mean and standard deviation of the errors in the four estimators of $\beta^{*}$ under N=100 simulations, with n=100. The quasi-optimal $\lambda_{2}$ is c=0.002 for Elastic-net, while c=0.027 is for the CSDE.

	*W*	$\lambda_{1}$	$L_{1}$ Error	TV Error
Lasso	81	0.065	2.133 (2.467)	1.137 (1.115)
Elastic-net			2.061 (1.439)	1.114 (0.805)
SPADES		0.053	1.922 (2.211)	1.258 (1.296)
CSDE			2.191 (4.812)	1.405 (2.329)
Lasso	131	0.068	2.032 (0.985)	1.352 (0.712)
Elastic-net			2.236 (2.498)	1.409 (1.056)
SPADES		0.056	1.880 (2.644)	0.972 (1.204)
CSDE			1.635 (0.342)	0.863 (0.402)
Lasso	211	0.071	2.572 (4.187)	1.605 (2.702)
Elastic-net			2.061 (1.883)	1.353 (1.516)
SPADES		0.058	1.764 (1.041)	0.832 (0.610)
CSDE			1.648 (0.168)	0.791 (0.415)
Lasso	321	0.074	2.120 (2.842)	1.146 (1.115)
Elastic-net			10.173 (82.753)	7.839 (67.887)
SPADES		0.061	2.106 (4.816)	0.818 (1.565)
CSDE			1.623 (0.085)	0.634 (0.199)

**Table 2:** The simulation result in Section 4.3. The mean and standard deviation of the errors in the four estimators of $\beta^{*}$ under N=100 simulations. The $\lambda_{2}$ is chosen as c=0.005 for Elastic-net, while c=0.203 for the CSDE.

	*W*	$\lambda_{1}$	$L_{1}$ Error	TV Error
Lasso	81	0.048	1.796 (0.006)	0.002 (0.001)
Elastic-net			1.796 (0.006)	0.002 (0.001)
SPADES		0.138	1.811 (0.013)	0.002 (0.005)
CSDE			1.806 (0.008)	0.003 (0.005)
Lasso	131	0.051	1.828 (0.006)	0.003 (0.001)
Elastic-net			1.830 (0.009)	0.004 (0.002)
SPADES		0.145	1.880 (0.006)	0.002 (0.005)
CSDE			1.854 (0.006)	0.002 (0.004)
Lasso	211	0.053	1.935 (0.010)	0.005 (0.003)
Elastic-net			2.061 (0.014)	0.007 (0.008)
SPADES		0.152	1.935 (0.008)	0.005 (0.003)
CSDE			1.861 (0.005)	0.003 (0.002)
Lasso	321	0.055	1.927 (0.031)	0.005 (0.002)
Elastic-net			2.123 (0.026)	0.009 (0.009)
SPADES		0.158	1.938 (0.008)	0.005 (0.003)
CSDE			1.852 (0.002)	0.002 (0.001)

**Table 3:** The low-dimensional simulation result in Section 4.4.

		$L_{1}$ Error	TV Error
Scenario 1	EM	0.255 (0.122)	0.205 (0.098)
	CSDE	0.206 (0.145)	0.185 (0.104)
Scenario 2	EM	0.111 (0.055)	0.111 (0.055)
	CSDE	0.109 (0.037)	0.108 (0.037)

## Data Availability

The authors would like to thank Song Xi Chen’s Group https://songxichen.com/(accessed on 20 December 2021) for sharing the meteorological dataset.

## Appendix A

For convenience, we first give a preliminary lemma and proof. Define the random variables $M_{j}=\frac{1}{n}\sum_{i=1}^{n}\left\{h_{j}\left(X_{i}\right)-Eh_{j}\left(X_{i}\right)\right\}.$ Consider the event E by $E={⋂}_{j=1}^{W}\left\{2\right|M_{j}\left|\leq {\tilde{\omega }}_{j}\right\}$, where ${\tilde{\omega }}_{k}:=2\sqrt{2}L_{k}\sqrt{\frac{1}{n}\log\frac{W}{\delta /2}}=:2\sqrt{2}L_{k}v\left(\delta /2\right)$. Then, we have the following lemma, which is cornerstone for the proofs in below.

**Lemma** **A1.**
*Suppose $\max_{1\leq j\leq W}L_{j}<\infty$ and $c=\frac{\min_{1\leq j\leq W}\left\{{\tilde{\omega }}_{j}\right\}}{B}$, for any $\beta \in R^{W}$ on the event E, we have ${∥h_{\hat{\beta }}-h∥}^{2}+\sum_{j=1}^{W}{\tilde{\omega }}_{j}|{\hat{\beta }}_{j}-\beta_{j}|+\sum_{j=1}^{W}c{\left({\hat{\beta }}_{j}-\beta_{j}\right)}^{2}\leq {∥h_{\beta }-h∥}^{2}+6\sum_{j\in I(\beta )}\omega_{j}\left|{\hat{\beta }}_{j}-\beta_{j}\right|.$*

### Appendix A.1. Proof of Lemma A1

According to the definition of $\hat{\beta }$, for any $\beta \in R^{W}$, we find $-\frac{2}{n}\sum_{i=1}^{n}h_{\hat{\beta }}\left(X_{i}\right)+{∥h_{\hat{\beta }}∥}^{2}+2\sum_{j=1}^{W}\omega_{j}|{\hat{\beta }}_{j}|+c\sum_{j=1}^{W}{\hat{\beta }}_{j}^{2}\leq -\frac{2}{n}\sum_{i=1}^{n}h_{\beta }\left(X_{i}\right)+{∥h_{\beta }∥}^{2}+2\sum_{j=1}^{W}\omega_{j}\left|\beta_{j}\right|+c\sum_{j=1}^{W}\beta_{j}^{2}.$ Then

$$\begin{array}{c} {∥h_{\hat{\beta }}∥}^{2}-{∥h_{\beta }∥}^{2}\leq \frac{2}{n}\sum_{i=1}^{n}h_{\hat{\beta }}\left(X_{i}\right)-\frac{2}{n}\sum_{i=1}^{n}h_{\beta }\left(X_{i}\right)+2\sum_{j=1}^{W}\omega_{j}|\beta_{j}|-2\sum_{j=1}^{W}\omega_{j}\left|{\hat{\beta }}_{j}\right|+c\sum_{j=1}^{W}\beta_{j}^{2}-c\sum_{j=1}^{W}{\hat{\beta }}_{j}^{2}. \end{array}$$

Note that

$$\begin{array}{cc} {∥h_{\hat{\beta }}-h∥}^{2} & ={∥h_{\hat{\beta }}-h_{\beta }+h_{\beta }-h∥}^{2}={∥h_{\hat{\beta }}-h_{\beta }∥}^{2}+{∥h_{\beta }-h∥}^{2}+2<h_{\beta }-h,h_{\hat{\beta }}-h_{\beta }>\\ & ={∥h_{\beta }-h∥}^{2}-2<h,h_{\hat{\beta }}-h_{\beta }>+2<h_{\beta },h_{\hat{\beta }}-h_{\beta }>+{∥h_{\hat{\beta }}-h_{\beta }∥}^{2}\\ & ={∥h_{\beta }-h∥}^{2}-2<h,h_{\hat{\beta }}-h_{\beta }>+{∥h_{\hat{\beta }}∥}^{2}-{∥h_{\beta }∥}^{2}. \end{array}$$

Combining the two result above, we obtain

(A1) $$\begin{array}{cc} {∥h_{\hat{\beta }}-h∥}^{2} & \leq {∥h_{\beta }-h∥}^{2}+2\sum_{j=1}^{W}\omega_{j}|\beta_{j}|-2\sum_{j=1}^{W}\omega_{j}\left|{\hat{\beta }}_{j}\right|+c\sum_{j=1}^{W}\beta_{j}^{2}-c\sum_{j=1}^{W}{\hat{\beta }}_{j}^{2}\\ & -2<h,h_{\hat{\beta }}-h_{\beta }>+\frac{2}{n}\sum_{i=1}^{n}h_{\hat{\beta }}\left(X_{i}\right)-\frac{2}{n}\sum_{i=1}^{n}h_{\beta }\left(X_{i}\right). \end{array}$$

According to the definition of $h_{\beta }\left(x\right)$, it gives $h_{\beta }\left(x\right)=\sum_{j=1}^{W}\beta_{j}h_{j}\left(x\right)$ with $\beta =(\beta_{1},\cdots ,\beta_{W})$. For the three terms in Equation (A1), we have

$$\begin{array}{cc} & -2<h,h_{\hat{\beta }}-h_{\beta }>+\frac{2}{n}\sum_{i=1}^{n}h_{\hat{\beta }}\left(X_{i}\right)-\frac{2}{n}\sum_{i=1}^{n}h_{\beta }\left(X_{i}\right)\\ = & 2\cdot \frac{1}{n}\sum_{i=1}^{n}\left(\sum_{j=1}^{W}{\hat{\beta }}_{j}h_{j}\left(X_{i}\right)-\sum_{j=1}^{W}\beta_{j}h_{j}\left(X_{i}\right)\right)-2E\left(h_{\beta^{\prime }}-h_{\beta }\right)\left(X_{i}\right){|}_{\beta^{\prime }=\hat{\beta }}\\ = & 2\sum_{j=1}^{W}\frac{1}{n}\sum_{i=1}^{n}h_{j}\left(X_{i}\right)\left({\hat{\beta }}_{j}-\beta_{j}\right)-2\sum_{j=1}^{W}E\left[h_{j}\left(X_{i}\right)\right]\left({\hat{\beta }}_{j}-\beta_{j}\right)\\ = & 2\sum_{j=1}^{W}\left(\frac{1}{n}\sum_{i=1}^{n}h_{j}\left(X_{i}\right)-E\left[h_{j}\left(X_{i}\right)\right]\right)\left({\hat{\beta }}_{j}-\beta_{j}\right). \end{array}$$

Then

$$\begin{array}{cc} {∥h_{\hat{\beta }}-h∥}^{2} & \leq {∥h_{\beta }-h∥}^{2}+2\sum_{j=1}^{W}\left(\frac{1}{n}\sum_{i=1}^{n}h_{j}\left(X_{i}\right)-E\left[h_{j}\left(X_{i}\right)\right]\right)\left({\hat{\beta }}_{j}-\beta_{j}\right)\\ & +2\sum_{j=1}^{W}\omega_{j}|\beta_{j}|-2\sum_{j=1}^{W}\omega_{j}\left|{\hat{\beta }}_{j}\right|+c\sum_{j=1}^{W}\beta_{j}^{2}-c\sum_{j=1}^{W}{\hat{\beta }}_{j}^{2}. \end{array}$$

Conditioning on E, we have ${∥h_{\hat{\beta }}-h∥}^{2}\leq {∥h_{\beta }-h∥}^{2}+\sum_{j=1}^{W}{\tilde{\omega }}_{j}|{\hat{\beta }}_{j}-\beta_{j}|+2\sum_{j=1}^{W}\omega_{j}\left(\right|\beta_{j}\left|-\right|{\hat{\beta }}_{j}\left|\right)+c\sum_{j=1}^{W}\left(\beta_{j}^{2}-{\hat{\beta }}_{j}^{2}\right).$ We add $\sum_{j=1}^{W}{\tilde{\omega }}_{j}\left|{\hat{\beta }}_{j}-\beta_{j}\right|+c\sum_{j=1}^{W}{\left(\beta_{j}-{\hat{\beta }}_{j}\right)}^{2}$ to both sides of the inequality, it gives

$$\begin{array}{cc} & {∥h_{\hat{\beta }}-h∥}^{2}+\sum_{j=1}^{W}{\tilde{\omega }}_{j}\left|{\hat{\beta }}_{j}-\beta_{j}\right|+c\sum_{j=1}^{W}{\left(\beta_{j}-{\hat{\beta }}_{j}\right)}^{2}\\ \leq & {∥h_{\beta }-h∥}^{2}+2\sum_{j=1}^{W}{\tilde{\omega }}_{j}|{\hat{\beta }}_{j}-\beta_{j}|+2\sum_{j=1}^{W}\omega_{j}\left(\right|\beta_{j}\left|-\right|{\hat{\beta }}_{j}\left|\right)+c\sum_{j=1}^{W}\left(\beta_{j}^{2}-{\hat{\beta }}_{j}^{2}\right)+c\sum_{j=1}^{W}{\left(\beta_{j}-{\hat{\beta }}_{j}\right)}^{2}. \end{array}$$

Note that

$$\begin{array}{cc} & c\left[\sum_{j=1}^{W}\left(\beta_{j}^{2}-{\hat{\beta }}_{j}^{2}\right)+\sum_{j=1}^{W}{\left(\beta_{j}-{\hat{\beta }}_{j}\right)}^{2}\right]=c\left[\sum_{j=1}^{W}\left(\beta_{j}^{2}-{\hat{\beta }}_{j}^{2}+\beta_{j}^{2}-2\beta_{j}{\hat{\beta }}_{j}+{\hat{\beta }}_{j}^{2}\right)\right]\\ = & 2c\sum_{j=1}^{W}\beta_{j}\left(\beta_{j}-{\hat{\beta }}_{j}\right)=2c\sum_{j\in I(\beta )}\beta_{j}\left(\beta_{j}-{\hat{\beta }}_{j}\right)\leq 2cB\sum_{j\in I(\beta )}|\beta_{j}-{\hat{\beta }}_{j}|\leq 2\sum_{j\in I(\beta )}{\tilde{\omega }}_{j}\left|\beta_{j}-{\hat{\beta }}_{j}\right|, \end{array}$$

where the last inequality is due to the assumption $c=\frac{\min_{1\leq j\leq W}\left\{{\tilde{\omega }}_{j}\right\}}{B}\leq \frac{{\tilde{\omega }}_{j}}{B}$. Thus, we obtain

$$\begin{array}{cc} & {∥h_{\hat{\beta }}-h∥}^{2}+\sum_{j=1}^{W}{\tilde{\omega }}_{j}\left|{\hat{\beta }}_{j}-\beta_{j}\right|+c\sum_{j=1}^{W}{\left({\hat{\beta }}_{j}-\beta_{j}\right)}^{2}\\ \leq & {∥h_{\beta }-h∥}^{2}+2\sum_{j=1}^{W}{\tilde{\omega }}_{j}|{\hat{\beta }}_{j}-\beta_{j}|+2\sum_{j=1}^{W}\omega_{j}\left(\right|\beta_{j}\left|-\right|{\hat{\beta }}_{j}\left|\right)+2\sum_{j\in I(\beta )}{\tilde{\omega }}_{j}\left|{\hat{\beta }}_{j}-\beta_{j}\right|\\ \leq & {∥h_{\beta }-h∥}^{2}+2\sum_{j=1}^{W}\omega_{j}|{\hat{\beta }}_{j}-\beta_{j}|+2\sum_{j=1}^{W}\omega_{j}\left(\right|\beta_{j}\left|-\right|{\hat{\beta }}_{j}\left|\right)+2\sum_{j\in I(\beta )}\omega_{j}\left|{\hat{\beta }}_{j}-\beta_{j}\right|, \end{array}$$

where the last inequality follows from ${\tilde{\omega }}_{j}\leq \omega_{j}$ for all *j*.

We know $\beta_{j}\neq 0$ if j∈I(β), and $\beta_{j}=0$ if j∉I(β). Considering $|\beta_{j}\left|-\right|{\hat{\beta }}_{j}\left|\leq \right|{\hat{\beta }}_{j}-\beta_{j}|$ for all *j*, we have $2\sum_{j=1}^{W}\omega_{j}|{\hat{\beta }}_{j}-\beta_{j}|+2\sum_{j=1}^{W}\omega_{j}\left(\right|\beta_{j}\left|-\right|{\hat{\beta }}_{j}\left|\right)\leq 4\sum_{j\in I(\beta )}\omega_{j}\left|{\hat{\beta }}_{j}-\beta_{j}\right|.$ Then

$$\begin{array}{cc} \; & ||h_{\hat{\beta }}{-h∥}^{2}+\sum_{j=1}^{W}{\tilde{\omega }}_{j}\left|{\hat{\beta }}_{j}-\beta_{j}\right|+c\sum_{j=1}^{W}{\left({\hat{\beta }}_{j}-\beta_{j}\right)}^{2}\\ \; & \leq ∥h_{\beta }{-h∥}^{2}+4\sum_{j\in I(\beta )}\omega_{j}|{\hat{\beta }}_{j}-\beta_{j}|+2\sum_{j\in I(\beta )}\omega_{j}\left|{\hat{\beta }}_{j}-\beta_{j}\right|\\ \; & =∥h_{\beta }{-h∥}^{2}+6\sum_{j\in I(\beta )}\omega_{j}\left|{\hat{\beta }}_{j}-\beta_{j}\right|. \end{array}$$

### Appendix A.2. Proof of Theorems

According to ${\tilde{\omega }}_{j}=2\sqrt{2}L_{j}\sqrt{\frac{1}{n}\log\frac{2W}{\delta }}$ in Equation (6), the sum of the independent random variables $\zeta_{ij}=h_{j}\left(X_{i}\right)-Eh_{j}\left(X_{i}\right)$ is determined by Hoeffding’s inequality, and $|h_{j}\left(X_{i}\right)|\leq 2L_{j}$. We obtain

$$\begin{array}{cc} P\left(E^{c}\right)=P\left(\overset{W}{\underset{j=1}{⋃}}\left\{2\right|M_{j}\left|>{\tilde{\omega }}_{j}\right\}\right) & \leq \sum_{j=1}^{W}P(2|M_{j}\left|>{\tilde{\omega }}_{j}\right)\leq 2\sum_{j=1}^{W}\exp\left(-\frac{2n^{2}\cdot {\tilde{\omega }}_{j}^{2}/4}{4nL_{j}^{2}}\right)\\ & =2\sum_{j=1}^{W}\exp\left(-\log\frac{2W}{\delta }\right)=2W\cdot \frac{\delta }{2W}=\delta . \end{array}$$

### Appendix A.3. Proof of Theorem 1

By Lemma A1, we need an upper bound on $\sum_{j\in I(\beta )}\omega_{j}\left|{\hat{\beta }}_{j}-\beta_{j}\right|$. For easy notation, let $q_{j}={\hat{\beta }}_{j}-\beta_{j},\;Q\left(\beta \right)=\sum_{j\in I(\beta )}|q_{j}|∥h_{j}∥,\;Q=\sum_{j=1}^{W}|q_{j}|∥h_{j}∥.$ According to the definition of H(β), that is, $H\left(\beta \right)=\max_{j\in I(\beta )}\frac{\omega_{j}}{v\left(\delta /2\right)∥h_{j}∥}$, we have

(A2) $$\sum_{j\in I(\beta )}\omega_{j}\left|{\hat{\beta }}_{j}-\beta_{j}\right|\leq v\left(\delta /2\right)H\left(\beta \right)Q\left(\beta \right).$$

Let $Q_{*}\left(\beta \right):=\sqrt{\sum_{j\in I(\beta )}q_{j}^{2}{∥h_{j}∥}^{2}}$. Using the definition of $h_{\beta }\left(x\right)$, we obtain $Q_{*}^{2}\left(\beta \right)=\sum_{j\in I(\beta )}q_{j}^{2}{∥h_{j}∥}^{2}={∥h_{\hat{\beta }}-h_{\beta }∥}^{2}-\sum_{i,j\notin I(\beta )}q_{i}q_{j}<h_{i},h_{j}>-\left(2\sum_{i\notin I(\beta )}\sum_{j\in I(\beta )}q_{i}q_{j}<h_{i},h_{j}>+\underset{i,j\in I(\beta ),i\neq j}{\sum \sum }q_{i}q_{j}<h_{i},h_{j}>\right).$ As i,j∉I(β), $\beta_{i}=\beta_{j}=0$, it is easy to see $\underset{i,j\notin I(\beta )}{\sum \sum }<h_{i},h_{j}>q_{i}q_{j}\geq 0$. Observe that

$$\begin{array}{cc} & 2\sum_{i\notin I(\beta )}\sum_{j\in I(\beta )}q_{i}q_{j}<h_{i},h_{j}>+\underset{i,j\in I(\beta ),i\neq j}{\sum \sum }q_{i}q_{j}<h_{i},h_{j}>\\ = & 2\sum_{i\notin I(\beta )}\sum_{j\in I(\beta )}q_{i}q_{j}<h_{i},h_{j}>+2\underset{i,j\in I(\beta ),j>i}{\sum \sum }q_{i}q_{j}<h_{i},h_{j}>=2\underset{i\in I(\beta ),j>i}{\sum \sum }q_{i}q_{j}<h_{i},h_{j}>. \end{array}$$

By the definitions of $\rho_{W}\left(i,j\right)$ and $\rho_{*}\left(\beta \right)$, then

$$\begin{array}{cc} Q_{*}^{2}\left(\beta \right) & \leq {∥h_{\hat{\beta }}-h_{\beta }∥}^{2}+2\underset{i\in I(\beta ),j>i}{\sum \sum }|q_{i}\left|\right|q_{j}|∥h_{i}∥∥h_{j}∥\frac{<h_{i},h_{j}>}{∥h_{i}∥∥h_{j}∥}\\ & \leq {∥h_{\hat{\beta }}-h_{\beta }∥}^{2}+2\rho_{*}\left(\beta \right)\max_{i\in I(\beta ),j>i}|q_{i}|∥h_{i}∥|q_{j}|∥h_{j}∥. \end{array}$$

By $\max_{i\in I(\beta )}|q_{i}|∥h_{i}∥\leq \sqrt{\sum_{j\in I(\beta )}q_{j}^{2}{∥h_{j}∥}^{2}}=Q_{*}\left(\beta \right),\;\max_{i\in I(\beta ),j>i}|q_{j}|∥h_{j}∥\leq \sum_{j=1}^{W}|q_{j}|∥h_{j}∥$,

(A3) $$\begin{array}{cc} Q_{*}^{2}\left(\beta \right) & \leq {∥h_{\hat{\beta }}-h_{\beta }∥}^{2}+2\rho_{*}\left(\beta \right)Q_{*}\left(\beta \right)\sum_{j=1}^{W}|q_{j}|∥h_{j}∥={∥h_{\hat{\beta }}-h_{\beta }∥}^{2}+2\rho_{*}\left(\beta \right)Q_{*}\left(\beta \right)Q. \end{array}$$

By Equation (A3), we can obtain $Q_{*}^{2}\left(\beta \right)-2\rho_{*}\left(\beta \right)Q_{*}\left(\beta \right)Q-{∥h_{\hat{\beta }}-h_{\beta }∥}^{2}\leq 0.$

To find the upper bound of $Q_{*}\left(\beta \right)$, applying the properties of the quadratic inequality to the above formula, we obtain that

(A4) $$\begin{array}{cc} Q_{*}\left(\beta \right)\leq \rho_{*}\left(\beta \right)Q+\sqrt{\rho_{*}^{2}\left(\beta \right)Q^{2}+{∥h_{\hat{\beta }}-h_{\beta }∥}^{2}} & \leq \rho_{*}\left(\beta \right)Q+[\rho_{*}\left(\beta \right)Q+∥h_{\hat{\beta }}-h_{\beta }∥]\\ & \leq 2\rho_{*}\left(\beta \right)Q+∥h_{\hat{\beta }}-h_{\beta }∥. \end{array}$$

Note that $W\left(\beta \right)=\left|I\left(\beta \right)\right|=\sum_{j=1}^{W}I\left(\beta_{j}\neq 0\right)$, employing the Cauchy–Schwarz inequalities, we have

$$\begin{array}{cc} W\left(\beta \right)\sum_{j\in I(\beta )}{\left|q_{j}\right|}^{2}{∥h_{j}∥}^{2} & =\sum_{j\in I(\beta )}I^{2}\left(j\in I\left(\beta \right)\right)\sum_{j\in I(\beta )}{\left|q_{j}\right|}^{2}{∥h_{j}∥}^{2}\\ & \geq {\left(\sum_{j\in I(\beta )}I(\{j\in I(\beta ))|q_{j}|∥h_{j}∥\right)}^{2}=Q^{2}\left(\beta \right). \end{array}$$

Then, $Q_{*}^{2}\left(\beta \right)=\sum_{j\in I(\beta )}{\left|q_{j}\right|}^{2}{∥h_{j}∥}^{2}\geq Q^{2}\left(\beta \right)/W\left(\beta \right).$ In combination with Equation (A4), we can obtain $Q\left(\beta \right)/\sqrt{W(\beta )}\leq Q_{*}\left(\beta \right)\leq 2\rho_{*}\left(\beta \right)Q+∥h_{\hat{\beta }}-h_{\beta }∥$. Therefore,

(A5) $$\begin{array}{c} Q\left(\beta \right)\leq 2\rho_{*}\left(\beta \right)\sqrt{W(\beta )}Q+\sqrt{W(\beta )}∥h_{\hat{\beta }}-h_{\beta }∥. \end{array}$$

By Lemma A1 and Equation (A2), we have the following inequality with probability exceeding 1−δ,

$$\begin{array}{cc} & {∥h_{\hat{\beta }}-h∥}^{2}+\sum_{j=1}^{W}{\tilde{\omega }}_{j}|{\hat{\beta }}_{j}-\beta_{j}|+\sum_{j=1}^{W}c{\left({\hat{\beta }}_{j}-\beta_{j}\right)}^{2}\leq {∥h_{\beta }-h∥}^{2}+6\sum_{j\in I(\beta )}\omega_{j}\left|{\hat{\beta }}_{j}-\beta_{j}\right|\\ \leq & {∥h_{\beta }-h∥}^{2}+6v\left(\delta /2\right)H\left(\beta \right)Q\left(\beta \right)\\ \leq & {∥h_{\beta }-h∥}^{2}+6v\left(\delta /2\right)H\left(\beta \right)[2\rho_{*}\left(\beta \right)\sqrt{W(\beta )}\sum_{j=1}^{W}|q_{j}|∥h_{j}∥+\sqrt{W(\beta )}∥h_{\hat{\beta }}-h_{\beta }∥]\;\;\left(\mathrm{by}\;\left(A5\right)\right)\\ = & {∥h_{\beta }-h∥}^{2}+12v\left(\delta /2\right)H\left(\beta \right)\rho_{*}\left(\beta \right)\sqrt{W(\beta )}\sum_{j=1}^{W}{\tilde{\omega }}_{j}\left|{\hat{\beta }}_{j}-\beta_{j}\right|\frac{∥h_{j}∥}{{\tilde{\omega }}_{j}}\\ + & 6v\left(\delta /2\right)H\left(\beta \right)\sqrt{W(\beta )}∥h_{\hat{\beta }}-h_{\beta }∥\\ \leq & {∥h_{\beta }-h∥}^{2}+12FH\left(\beta \right)\rho_{*}\left(\beta \right)\sqrt{W(\beta )}\sum_{j=1}^{W}{\tilde{\omega }}_{j}\left|{\hat{\beta }}_{j}-\beta_{j}\right|+6v\left(\delta /2\right)H\left(\beta \right)\sqrt{W(\beta )}∥h_{\hat{\beta }}-h_{\beta }∥\\ \leq & {∥h_{\beta }-h∥}^{2}+\gamma \sum_{j=1}^{W}{\tilde{\omega }}_{j}\left|{\hat{\beta }}_{j}-\beta_{j}\right|+6v\left(\delta /2\right)H\left(\beta \right)\sqrt{W(\beta )}∥h_{\hat{\beta }}-h_{\beta }∥, \end{array}$$

where the second last inequality follows from the definition of $F:=\max_{1\leq j\leq W}\frac{v\left(\delta /2\right)∥h_{j}∥}{{\tilde{\omega }}_{j}}$, and the last inequality is derived by the assumption $12FH\left(\beta \right)\rho_{*}\left(\beta \right)\sqrt{W(\beta )}\leq \gamma ,\left(0<\gamma \leq 1\right).$

Further, we can find that, with probability at least 1−δ,

$$\begin{array}{cc} & {∥h_{\hat{\beta }}-h∥}^{2}+\left(1-\gamma \right)\sum_{j=1}^{W}{\tilde{\omega }}_{j}\left|{\hat{\beta }}_{j}-\beta_{j}\right|+\sum_{j=1}^{W}c{\left({\hat{\beta }}_{j}-\beta_{j}\right)}^{2}\\ \leq & {∥h_{\beta }-h∥}^{2}+6v\left(\delta /2\right)H\left(\beta \right)\sqrt{W(\beta )}∥h_{\hat{\beta }}-h_{\beta }∥\\ = & {∥h_{\beta }-h∥}^{2}+6v\left(\delta /2\right)H\left(\beta \right)\sqrt{W(\beta )}∥h_{\hat{\beta }}-h+h-h_{\beta }∥\\ \leq & {∥h_{\beta }-h∥}^{2}+6v\left(\delta /2\right)H\left(\beta \right)\sqrt{W(\beta )}∥h_{\hat{\beta }}-h∥+6v\left(\delta /2\right)H\left(\beta \right)\sqrt{W(\beta )}∥h-h_{\beta }∥. \end{array}$$

Using the elementary inequality $2st\leq s^{2}/\alpha +\alpha t^{2}\;\left(s,t\in R,\alpha >1\right)$ to the last two terms of the above inequality, it yields

$$\begin{array}{c} 2\left\{3v\left(\delta /2\right)H\left(\beta \right)\sqrt{W(\beta )}\right\}∥h_{\hat{\beta }}-h∥\leq \alpha \cdot 9v^{2}\left(\delta /2\right)H^{2}\left(\beta \right)W\left(\beta \right)+{∥h_{\hat{\beta }}-h∥}^{2}/\alpha ,\\ 2\left\{3v\left(\delta /2\right)H\left(\beta \right)\sqrt{W(\beta )}\right\}∥h_{\beta }-h∥\leq \alpha \cdot 9v^{2}\left(\delta /2\right)H^{2}\left(\beta \right)W\left(\beta \right)+{∥h_{\beta }-h∥}^{2}/\alpha . \end{array}$$

Thus,

$$\begin{array}{cc} & {∥h_{\hat{\beta }}-h∥}^{2}+\left(1-\gamma \right)\sum_{j=1}^{W}{\tilde{\omega }}_{j}\left|{\hat{\beta }}_{j}-\beta_{j}\right|+\sum_{j=1}^{W}c{\left({\hat{\beta }}_{j}-\beta_{j}\right)}^{2}\\ \leq & {∥h_{\beta }-h∥}^{2}+18\alpha v^{2}\left(\delta /2\right)H^{2}\left(\beta \right)W\left(\beta \right)+{∥h_{\hat{\beta }}-h∥}^{2}/\alpha +{∥h_{\beta }-h∥}^{2}/\alpha . \end{array}$$

Simplifying, we have

(A6) $$\begin{array}{cc} & {∥h_{\hat{\beta }}-h∥}^{2}+\frac{\alpha (1-\gamma )}{\left(\alpha -1\right)}\sum_{j=1}^{W}{\tilde{\omega }}_{j}\left|{\hat{\beta }}_{j}-\beta_{j}\right|+\frac{\alpha }{\alpha -1}\sum_{j=1}^{W}c{\left({\hat{\beta }}_{j}-\beta_{j}\right)}^{2}\\ \; & \leq \frac{\alpha +1}{\alpha -1}{∥h_{\beta }-h∥}^{2}+\frac{18\alpha^{2}}{\alpha -1}H^{2}\left(\beta \right)v^{2}\left(\delta /2\right)W\left(\beta \right),\;\alpha >1,0<\gamma \leq 1. \end{array}$$

Optimizing α to obtain the sharp upper bounds for the above oracle inequality

$$\begin{array}{cc} \alpha_{opt1}: & =\underset{\alpha >1}{\arg\min}\left\{\frac{\alpha +1}{\alpha -1}{∥h_{\beta }-h∥}^{2}+\frac{18\alpha^{2}}{\alpha -1}H^{2}\left(\beta \right)v^{2}\left(\delta /2\right)W\left(\beta \right)\right\}\\ & =1+\sqrt{1+\frac{{∥h_{\beta }-h∥}^{2}}{9H^{2}\left(\beta \right)v^{2}\left(\delta /2\right)W\left(\beta \right)}} \end{array}$$

by the first order condition. To date, Theorem 1 is proved by substituting $\alpha_{opt1}$ into Equation (A6).

### Appendix A.4. Proof of Theorem 2

By the minimal eigenvalue assumption for $\psi_{W}$, we have

(A7) $$\begin{array}{c} {∥h_{\beta }∥}^{2}={||\sum_{j=1}^{W}\beta_{j}h_{j}\left(x\right)||}^{2}=\beta^{T}\psi_{W}\beta \geq \lambda_{W}{∥\beta ∥}^{2}\geq \lambda_{W}\sum_{j\in I(\beta )}\beta_{j}^{2}. \end{array}$$

Using the definition of $\omega_{j}$ and assumption $L_{\min}:=\min_{1\leq j\leq W}L_{j}>0$,

$$\omega_{j}=2L_{j}\left(\sqrt{\frac{2\log(2W/\delta )}{n}}+\frac{cB}{2L_{j}}\right)\leq 2L_{j}\left(\sqrt{\frac{2\log(2W/\delta )}{n}}+\frac{cB}{2L_{\min}}\right).$$

Since $cB={\tilde{\omega }}_{\min}=2\sqrt{2}L_{\min}v\left(\delta /2\right)$ and $v\left(\delta /2\right)=\sqrt{\frac{\log(2W/\delta )}{n}}$, we have $\omega_{j}\leq 4\sqrt{2}L_{j}v\left(\delta /2\right).$

Let $G\left(\beta \right)=\sum_{j\in I(\beta )}L_{j}^{2}$, by the Cauchy–Schwartz inequality, we obtain

(A8) $$\begin{array}{cc} 6 & \sum_{j\in I(\beta )}\omega_{j}|{\hat{\beta }}_{j}-\beta_{j}|\leq 24\sqrt{2}v\left(\delta /2\right)\sum_{j\in I(\beta )}L_{j}\left|{\hat{\beta }}_{j}-\beta_{j}\right|\\ & \leq 24\sqrt{2}v\left(\delta /2\right)\sqrt{\sum_{j\in I(\beta )}L_{j}^{2}}\sqrt{\sum_{j\in I(\beta )}{\left({\hat{\beta }}_{j}-\beta_{j}\right)}^{2}}\leq 24\sqrt{2}v\left(\delta /2\right)\sqrt{\frac{G(\beta )}{\lambda_{W}}}∥h_{\hat{\beta }}-h_{\beta }∥, \end{array}$$

where the last inequality above is from Equation (A7) due to

$${∥h_{\hat{\beta }}-h_{\beta }∥}^{2}=\underset{1\leq i,j\leq W}{\sum \sum }\left({\hat{\beta }}_{i}-\beta_{i}\right)\left({\hat{\beta }}_{j}-\beta_{j}\right)<h_{i},h_{j}>\geq \lambda_{W}\sum_{j\in I(\beta )}{\left({\hat{\beta }}_{j}-\beta_{j}\right)}^{2}.$$

Let $b\left(\beta \right):=12\sqrt{2}v\left(\delta /2\right)\sqrt{\frac{G(\beta )}{\lambda_{W}}}$, Lemma 2 implies

$$\begin{array}{cc} & {∥h_{\hat{\beta }}-h∥}^{2}+\sum_{j=1}^{W}{\tilde{\omega }}_{j}|{\hat{\beta }}_{j}-\beta_{j}|+\sum_{j=1}^{W}c{\left({\hat{\beta }}_{j}-\beta_{j}\right)}^{2}\leq {∥h_{\beta }-h∥}^{2}+2b\left(\beta \right)∥h_{\hat{\beta }}-h_{\beta }∥\\ = & {∥h_{\beta }-h∥}^{2}+2b\left(\beta \right)(∥h_{\hat{\beta }}-h+h-h_{\beta }∥)\leq {∥h_{\beta }-h∥}^{2}+2b\left(\beta \right)∥h_{\hat{\beta }}-h∥+2b\left(\beta \right)∥h_{\beta }-h∥. \end{array}$$

Using the inequality $2st\leq s^{2}/\alpha +\alpha t^{2}$(s,t∈R,α>1) for the last two terms on the right side of the above inequality, we find

$$\begin{array}{cc} 2b\left(\beta \right)∥h_{\hat{\beta }}-h∥+2b(\beta )∥h_{\beta }-h∥ & \leq {∥h_{\hat{\beta }}-h∥}^{2}/\alpha +b^{2}\left(\beta \right)\alpha +{∥h_{\beta }-h∥}^{2}/\alpha +b^{2}\left(\beta \right)\alpha \\ & ={∥h_{\hat{\beta }}-h∥}^{2}/\alpha +{∥h_{\beta }-h∥}^{2}/\alpha +2b^{2}\left(\beta \right)\alpha . \end{array}$$

Thus,

$$\begin{array}{cc} {∥h_{\hat{\beta }}-h∥}^{2}+\sum_{j=1}^{W}{\tilde{\omega }}_{j}\left|{\hat{\beta }}_{j}-\beta_{j}\right|+\sum_{j=1}^{W}c{\left({\hat{\beta }}_{j}-\beta_{j}\right)}^{2}\; & \leq {∥h_{\beta }-h∥}^{2}+{∥h_{\hat{\beta }}-h∥}^{2}/\alpha \\ & +{∥h_{\beta }-h∥}^{2}/\alpha +2b^{2}\left(\beta \right)\alpha \end{array}$$

gives $\frac{\alpha -1}{\alpha }{∥h_{\hat{\beta }}-h∥}^{2}+\sum_{j=1}^{W}{\tilde{\omega }}_{j}|{\hat{\beta }}_{j}-\beta_{j}|+\sum_{j=1}^{W}c{\left({\hat{\beta }}_{j}-\beta_{j}\right)}^{2}\leq \frac{\alpha +1}{\alpha }{∥h_{\beta }-h∥}^{2}+2\alpha b^{2}\left(\beta \right)$. Therefore,

$$\begin{array}{c} {∥h_{\hat{\beta }}-h∥}^{2}+\frac{\alpha }{\alpha -1}\sum_{j=1}^{W}{\tilde{\omega }}_{j}\left|{\hat{\beta }}_{j}-\beta_{j}\right|+\frac{\alpha }{\alpha -1}\sum_{j=1}^{W}c{\left({\hat{\beta }}_{j}-\beta_{j}\right)}^{2}\\ \leq \frac{\alpha +1}{\alpha -1}{∥h_{\beta }-h∥}^{2}+\frac{2\alpha^{2}}{\alpha -1}b^{2}\left(\beta \right)\\ =\frac{\alpha +1}{\alpha -1}{∥h_{\beta }-h∥}^{2}+\frac{576\alpha^{2}}{\alpha -1}\frac{G(\beta )}{\lambda_{W}}v^{2}\left(\delta /2\right). \end{array}$$

To obtain the sharp upper bounds for the above oracle inequality, we optimize α

$$\begin{array}{cc} \alpha_{opt2}: & =\underset{\alpha >1}{\arg\min}\left\{\frac{\alpha +1}{\alpha -1}{∥h_{\beta }-h∥}^{2}+\frac{576\alpha^{2}}{\alpha -1}\frac{G(\beta )}{\lambda_{W}}v^{2}\left(\delta /2\right)\right\}=1+\sqrt{1+\frac{{∥h_{\beta }-h∥}^{2}}{288\frac{G(\beta )}{\lambda_{W}}v^{2}\left(\delta /2\right)}}, \end{array}$$

by the first-order condition. This completes the proof of Theorem 2.

### Appendix A.5. Proof of Corollory 1

Let ${\tilde{\omega }}_{\min}:=\min_{1\leq j\leq W}{\tilde{\omega }}_{j}$. We replace v(δ/2) in Theorem 1 by the larger value v(δ/2W). Substituting $\beta =\beta^{*}$ in Theorem 1, we have

$$\begin{array}{c} \frac{\alpha_{opt1}\left(1-\gamma \right)}{\alpha_{opt1}-1}\sum_{j=1}^{W}{\tilde{\omega }}_{j}\left|{\hat{\beta }}_{j}-\beta_{j}^{*}\right|\leq \frac{18\alpha_{opt1}^{2}}{\alpha_{opt1}-1}H^{2}\left(\beta^{*}\right)v^{2}\left(\delta /2W\right)W\left(\beta^{*}\right) \end{array}$$

by $h=h_{\beta^{*}}$. Since ${\tilde{\omega }}_{j}\geq {\tilde{\omega }}_{\min}$ for all *j*, we obtain

$$\begin{array}{c} \sum_{j=1}^{W}\left|{\hat{\beta }}_{j}-\beta_{j}^{*}\right|\leq \frac{18\alpha_{opt1}}{1-\gamma }\cdot \frac{1}{{\tilde{\omega }}_{\min}}\cdot \max_{j\in I(\beta )}\frac{\omega_{j}^{2}}{{∥h_{j}∥}^{2}}\cdot W\left(\beta^{*}\right). \end{array}$$

In this case, $\alpha_{opt1}=2$, and $∥h_{j}∥=1$; thus,

$$\begin{array}{cc} ∥\hat{\beta }-\beta^{*}∥ & \leq \frac{36}{1-\gamma }\cdot \max_{j\in I(\beta )}\frac{\omega_{j}^{2}}{{\tilde{\omega }}_{\min}}\cdot W\left(\beta^{*}\right)\\ & =\frac{72\sqrt{2}v\left(\delta /2W\right)W\left(\beta^{*}\right)}{1-\gamma }\max_{j\in I(\beta )}\frac{{\left(L_{j}+L_{\min}\right)}^{2}}{L_{\min}}\leq \frac{72\sqrt{2}v\left(\delta /2W\right)W\left(\beta^{*}\right)}{1-\gamma }\frac{{\left(L+L_{\min}\right)}^{2}}{L_{\min}} \end{array}$$

from ${\tilde{\omega }}_{\min}=2\sqrt{2}v\left(\delta /2W\right)L_{\min}$ and

$\omega_{j}^{2}={\left[2\sqrt{2}v\left(\delta /2W\right)\right]}^{2}{\left[L_{j}+\frac{{\tilde{\omega }}_{\min}}{2\sqrt{2}v\left(\delta /2W\right)}\right]}^{2}={\left[2\sqrt{2}v\left(\delta /2W\right)\right]}^{2}{\left[L_{j}+L_{\min}\right]}^{2}.$ This completes the proof of Corollary 1.

### Appendix A.6. Proof of Corollary 2

Let $\beta =\beta^{*}$ in Theorem 2, with $\alpha_{opt2}=2$, we replace v(δ/2) in Theorem 2 by the larger value v(δ/2W), then $\sum_{j=1}^{W}{\tilde{\omega }}_{\min}|{\hat{\beta }}_{j}-\beta_{j}^{*}|\leq \sum_{j=1}^{W}{\tilde{\omega }}_{j}\left|{\hat{\beta }}_{j}-\beta_{j}^{*}\right|\leq \frac{576\alpha_{opt2}G^{*}}{\lambda_{W}}v^{2}\left(\delta /2W\right).$ By the definition of ${\tilde{\omega }}_{\min}$, we can obtain

$$\sum_{j=1}^{W}\left|{\hat{\beta }}_{j}-\beta_{j}^{*}\right|\leq \frac{576\alpha_{opt2}G^{*}v^{2}\left(\delta /2\right)}{{\tilde{\omega }}_{\min}\lambda_{W}}=\frac{576\cdot 2G^{*}v^{2}\left(\delta /2W\right)}{2\sqrt{2}v\left(\delta /2W\right)L_{\min}\lambda_{W}}=\frac{288\sqrt{2}G^{*}v\left(\delta /2W\right)}{L_{\min}\lambda_{W}}.$$

This concludes the proof of Corollary 2.

### Appendix A.7. Proof of Theorem 3

The following lemma is by virtue of the KKT conditions. It derives a bound of P(I*⫅I^), which is easily analyzed.

**Lemma** **A2** (Proposition 3.3 in [33])**.** *P(I*⫅I^)≤W(β*)maxk∈I*P(β^k=0andβk*≠0).*

To present the proof of Theorem 3, we first notice that P(I^≠I*)≤P(I*⫅I^)+P(I^⫅I*). Next, we control the probability on the right side of the above inequality.

For the control of P(I*⫅I^), by Lemma A2, it remains to bound $P({\hat{\beta }}_{k}=0\;\mathrm{and}\;\beta_{k}^{*}\neq 0)$.

Below, we will use the conclusion of Lemma 2 (KKT conditions). Recall that $E\left[h_{k}\left(Z_{1}\right)\right]=\sum_{j\in I_{*}}\beta_{j}^{*}<h_{k},h_{j}>=\sum_{j=1}^{W}\beta_{j}^{*}<h_{k},h_{j}>$. Since we assume that the density of $Z_{1}$ is the mixture density $h_{\beta^{*}}=\sum_{j\in I_{*}}\beta_{j}^{*}h_{j}$. Therefore, for $k\in I_{*}$, we have,

P(β^k=0andβk*≠0)=P1n∑i=1nhk(Xi)−∑j=1Wβ^j<hj,hk>≤22v(δ/2W)Lk;βk*≠0=P1n∑i=1nhk(Xi)−E[hk(Z1)]+E[hk(Z1)]−∑j=1Wβ^j<hj,hk>≤22v(δ/2W)Lk;βk*≠0=P1n∑i=1nhk(Xi)−E[hk(Z1)]−∑j=1W(β^j−βj*)<hj,hk>≤22v(δ/2W)Lk;βk*≠0=P1n∑i=1nhk(Xi)−E[hk(Z1)]−∑j≠kW(β^j−βj*)<hj,hk>+βk*∥hk∥2≤22v(δ/2W)Lk≤P|βk*∥hk∥2−22v(δ/2W)Lk≤1n∑i=1nhk(Xi)−E[hk(Z1)]+∑j≠kW(β^j−βj*)<hj,hk>(A9)≤P1n∑i=1nhk(Xi)−E[hk(Z1)]≥|βk*|∥hk∥22−2v(δ/2W)Lk(A10)+P∑j≠kW(β^j−βj*)<hj,hk>≥|βk*|∥hk∥22−2v(δ/2W)Lk.

Similar to Lemma 2, for Equation (A9), we use Hoeffding’s inequality. Since $∥h_{k}∥=1$ for all *k*. Put $\epsilon_{k}:=\left|E\left[h_{k}\left(X_{1}\right)\right]-E\left[h_{k}\left(Z_{1}\right)\right]\right|$. Consider Condition (B), $\min_{k\in I_{*}}\left|\beta_{k}^{*}\right|\geq 4\sqrt{2}v\left(\delta /2W\right)L$ and $L\geq \max_{1\leq k\leq W}L_{k}$, then we have

(A11) $$\begin{array}{cc} P & \left(\left|\frac{1}{n}\sum_{i=1}^{n}h_{k}\left(X_{i}\right)-E\left[h_{k}\left(Z_{1}\right)\right]\right|\geq \frac{|\beta_{k}^{*}|{∥h_{k}∥}^{2}}{2}-\sqrt{2}v\left(\delta /2W\right)L_{k}\right)\\ & =P\left(\left|\frac{1}{n}\sum_{i=1}^{n}h_{k}\left(X_{i}\right)-E\left[h_{k}\left(X_{1}\right)\right]+E\left[h_{k}\left(X_{1}\right)\right]-E\left[h_{k}\left(Z_{1}\right)\right]\right|\geq \frac{|\beta_{k}^{*}|{∥h_{k}∥}^{2}}{2}-\sqrt{2}v\left(\delta /2W\right)L_{k}\right)\\ & \leq P\left(\left|\frac{1}{n}\sum_{i=1}^{n}h_{k}\left(X_{i}\right)-E\left[h_{k}\left(X_{1}\right)\right]\right|\geq \frac{|\beta_{k}^{*}|}{2}-\sqrt{2}v\left(\delta /2W\right)L-\epsilon_{k}\right)\\ & \leq P\left(\left|\frac{1}{n}\sum_{i=1}^{n}h_{k}\left(X_{i}\right)-E\left[h_{k}\left(X_{1}\right)\right]\right|\geq 2\sqrt{2}v\left(\delta /2W\right)L-\sqrt{2}v\left(\delta /2W\right)L-\epsilon_{k}\right)\\ & =P\left(\left|\frac{1}{n}\sum_{i=1}^{n}h_{k}\left(X_{i}\right)-E\left[h_{k}\left(X_{1}\right)\right]\right|\geq \sqrt{2}v\left(\delta /2W\right)L-\epsilon_{k}\right)\\ & =P\left(\left|\frac{1}{n}\sum_{i=1}^{n}h_{k}\left(X_{i}\right)-E\left[h_{k}\left(X_{1}\right)\right]\right|\geq \sqrt{2}v\left(\delta /2W\right)L\left(1-\epsilon_{k}^{*}\right)\right)\;\;\left(\mathrm{let}\;\epsilon_{k}^{*}=\epsilon_{k}/\sqrt{2}v\left(\delta /2W\right)L\right)\\ & \leq 2\exp\left\{-\frac{4n^{2}v^{2}\left(\delta /2W\right)L^{2}{\left(1-\epsilon_{k}^{*}\right)}^{2}}{4nL^{2}}\right\}\\ & =2\exp\left\{-n{\left(1-\epsilon_{k}^{*}\right)}^{2}\frac{\log(2W^{2}/\delta )}{n}\right\}=2{\left(\frac{\delta }{2W^{2}}\right)}^{{\left(1-\epsilon_{k}^{*}\right)}^{2}}. \end{array}$$

For the upper bound of Equation (A10), using Condition (A) and Condition (B), by the definitions of $\rho_{*}\left(\beta^{*}\right)$ and $W(\beta^{*})$, we obtain

$$\begin{array}{cc} & P\left(\left|\sum_{j\neq k}^{W}\left({\hat{\beta }}_{j}-\beta_{j}^{*}\right)<h_{j},h_{k}>\right|\geq \frac{|\beta_{k}^{*}|{∥h_{k}∥}^{2}}{2}-\sqrt{2}v\left(\delta /2W\right)L_{k}\right)\\ = & P\left(\left|\sum_{j\neq k}^{W}\left({\hat{\beta }}_{j}-\beta_{j}^{*}\right)<h_{j},h_{k}>\right|\geq \frac{|\beta_{k}^{*}|}{2}-\sqrt{2}v\left(\delta /2W\right)L_{k}\right)\\ \leq & P\left(\left|\sum_{j\neq k}^{W}\left({\hat{\beta }}_{j}-\beta_{j}^{*}\right)\frac{<h_{j},h_{k}>}{∥h_{j}∥∥h_{k}∥}\cdot ∥h_{j}∥∥h_{k}∥\right|\geq 2\sqrt{2}v\left(\delta /2W\right)L-\sqrt{2}v\left(\delta /2W\right)L\right)\\ \leq & P\left(\rho_{*}\left(\beta^{*}\right)\sum_{j\neq k}^{W}\left|{\hat{\beta }}_{j}-\beta_{j}^{*}\right|\geq \sqrt{2}v\left(\delta /2W\right)L\right)\leq P\left(\sum_{j=1}^{W}\left|{\hat{\beta }}_{j}-\beta_{j}^{*}\right|\geq \frac{\sqrt{2}v\left(\delta /2W\right)L}{\rho_{*}\left(\beta^{*}\right)}\right)\\ \leq & P\left(\sum_{j=1}^{W}\left|{\hat{\beta }}_{j}-\beta_{j}^{*}\right|\geq \frac{288\sqrt{2}G^{*}v\left(\delta /2W\right)}{L_{\min}\lambda_{W}}\right)\leq \frac{\delta }{W}. \end{array}$$

where the second last inequality is by Condition (A), and the last inequality above is by using the $\ell_{1}$-estimation oracle inequality in Corollary 2.

Therefore, by the definition of $W(\beta^{*})$, $W\left(\beta^{*}\right)=\left|I_{*}\right|\leq W$, we find

P(I*⫅I^)≤W(β*)maxk∈I*P(β^k=0)≤W(β*)2δ2W2(1−ϵk*)2+W(β*)δW≤2Wδ2W2(1−ϵk*)2+WδW=2Wδ2W2(1−ϵk*)2+δ.

For the control of P(I^⫅I*), let

(A12) $$\begin{array}{c} \tilde{\eta }=\underset{\eta \in R^{W(\beta^{*})}}{\arg\min}z\left(\eta \right), \end{array}$$

where $z\left(\eta \right)=-\frac{2}{n}\sum_{i=1}^{n}\sum_{j\in I_{*}}\eta_{j}h_{j}\left(X_{i}\right)+{∥\sum_{j\in I_{*}}\eta_{j}h_{j}∥}^{2}+\sum_{j\in I_{*}}\left(4\sqrt{2}v\left(\delta /2\right)L_{j}+2cB\right)\left|\eta_{j}\right|+c\sum_{j\in I_{*}}\eta_{j}^{2}.$ Consider the following random event

(A13) $$\begin{array}{cc} \underset{k\notin I_{*}}{⋂} & \left\{\left|-\frac{1}{n}\sum_{i=1}^{n}h_{k}\left(X_{i}\right)+\sum_{j\in I_{*}}{\tilde{\eta }}_{j}<h_{j},h_{k}>\right|\leq 2\sqrt{2}v\left(\delta /2\right)L_{k}\right\}\\ & \subseteq \underset{k\notin I_{*}}{⋂}\left\{\left|-\frac{1}{n}\sum_{i=1}^{n}h_{k}\left(X_{i}\right)+\sum_{j\in I_{*}}{\tilde{\eta }}_{j}<h_{j},h_{k}>\right|\leq 2\sqrt{2}v\left(\delta /2W\right)L\right\}:=\Psi . \end{array}$$

Let $\bar{\eta }\in R^{W}$ be a vector corresponding to the component of the index set $I_{*}$ having $\tilde{\eta }$ given by Equation (A12), and the component at other corresponding positions is 0. By Lemma 1, we know that $\bar{\eta }\in R^{W}$ is a solution of Equation (3) on the event Ψ. It is recalled that $\hat{\beta }\in R^{W}$, which is also a solution of Equation (3). Through the definition of the indicator set $\hat{I}$, we have ${\hat{\beta }}_{k}\neq 0$ for $k\in \hat{I}$. By construction, we obtain ${\tilde{\eta }}_{k}\neq 0$ for some subset $T⫅I_{*}$. The KKT conditions indicate that any two solutions have non-zero components at the same positions. Therefore, $\hat{I}=T⫅I_{*}$ on the event Ψ. Further, we can write

P(I^⊈I*)≤P(Ψc)=P⋃k∉I*−1n∑i=1nhk(Xi)+∑j∈I*η˜j<hj,hk>≥22v(δ/2W)L≤∑k∉I*P−1n∑i=1nhk(Xi)+∑j∈I*η˜j<hj,hk>≥22v(δ/2W)L=∑k∉I*P−1n∑i=1nhk(Xi)+E[hk(Z1)]−E[hk(Z1)]+∑j∈I*η˜j<hj,hk>≥22v(δ/2W)L=∑k∉I*P1n∑i=1nhk(Xi)−E[hk(Z1)]−∑j∈I*(η˜j−βj*)<hj,hk>≥22v(δ/2W)L(A14)≤∑k∉I*P1n∑i=1nhk(Xi)−E[hk(Z1)]≥2v(δ/2W)L(A15)+∑k∉I*P∑j∈I*|η˜j−βj*|<hj,hk>≥2v(δ/2W)L.

According to the previously proven Formula (A11), we find

$$\begin{array}{cc} & \sum_{k\notin I_{*}}P\left\{\left|\frac{1}{n}\sum_{i=1}^{n}h_{k}\left(Z_{i}\right)-Eh_{k}\left(Z_{1}\right)\right|\geq \sqrt{2}v\left(\delta /2W\right)L\right\}\\ \leq & \sum_{k\notin I_{*}}P\left\{\left|\frac{1}{n}\sum_{i=1}^{n}h_{k}\left(X_{i}\right)-Eh_{k}\left(X_{1}\right)\right|\geq \sqrt{2}v\left(\delta /2W\right)L-\epsilon_{k}\right\}\\ = & \sum_{k=1}^{W}P\left\{\left|\frac{1}{n}\sum_{i=1}^{n}h_{k}\left(X_{i}\right)-Eh_{k}\left(X_{1}\right)\right|\geq \sqrt{2}v\left(\delta /2W\right)L\left(1-\epsilon_{k}^{*}\right)\right\}\leq 2W{\left(\frac{\delta }{2W^{2}}\right)}^{{\left(1-\epsilon_{k}^{*}\right)}^{2}}. \end{array}$$

For the upper bound of Equation (A15), observe Theorem 2, we can use a larger v(δ/2W) instead of v(δ/2). Consider the construction of $\tilde{\eta }$ in Equation (A12), we obtain

$$\begin{array}{c} P\left(\sum_{j\in I_{*}}\left|{\tilde{\eta }}_{j}-\beta_{j}^{*}\right|\geq \frac{288\sqrt{2}G^{*}v\left(\delta /2W\right)}{L_{\min}\lambda_{W}}\right)\leq \frac{\delta }{W}. \end{array}$$

Similarly, we have

$$\begin{array}{cc} & \sum_{k\notin I_{*}}P\left\{\sum_{j\in I_{*}}\left|{\tilde{\eta }}_{j}-\beta_{j}^{*}\right|\left|<h_{j},h_{k}>\right|\geq \sqrt{2}v\left(\delta /2W\right)L\right\}\\ \leq & \sum_{k=1}^{W}P\left\{\sum_{j\in I_{*}}\left|{\tilde{\eta }}_{j}-\beta_{j}^{*}\right|\left|\frac{<h_{j},h_{k}>}{∥h_{j}∥∥h_{k}∥}∥h_{j}∥∥h_{k}∥\right|\geq \sqrt{2}v\left(\delta /2W\right)L\right\}\\ \leq & \sum_{k=1}^{W}P\left\{\sum_{j\in I_{*}}\left|{\tilde{\eta }}_{j}-\beta_{j}^{*}\right|\rho_{*}\left(\beta^{*}\right)\geq \sqrt{2}v\left(\delta /2W\right)L\right\}\\ = & \sum_{k=1}^{W}P\left\{\sum_{j\in I_{*}}\left|{\tilde{\eta }}_{j}-\beta_{j}^{*}\right|\geq \frac{\sqrt{2}v\left(\delta /2W\right)L}{\rho_{*}\left(\beta^{*}\right)}\right\}\\ (\mathrm{using}\;\mathrm{Condition}\;\left(A\right))\; & \leq \sum_{k=1}^{W}P\left\{\sum_{j\in I_{*}}\left|{\tilde{\eta }}_{j}-\beta_{j}^{*}\right|\geq \frac{288\sqrt{2}G^{*}v\left(\delta /2W\right)}{L_{\min}\lambda_{W}}\right\}\leq \sum_{k=1}^{W}\frac{\delta }{W}=\delta . \end{array}$$

Combining all the bounds above, we can obtain

P(I^≠I*)≤P(I*⫅I^)+P(I^⫅I*)≤2Wδ2W2(1−ϵk*)2+δ+2Wδ2W2(1−ϵk*)2+δ=4Wδ2W2(1−ϵk*)2+2δ.

This completes the proof of Theorem 3.
